# The High‐Frequency Signature of Slow and Fast Laboratory Earthquakes

**DOI:** 10.1029/2022JB024170

**Published:** 2022-06-07

**Authors:** David C. Bolton, Srisharan Shreedharan, Gregory C. McLaskey, Jacques Rivière, Parisa Shokouhi, Daniel T. Trugman, Chris Marone

**Affiliations:** ^1^ University of Texas Institute for Geophysics Austin TX USA; ^2^ Department of Civil and Environmental Engineering Cornell University Ithaca NY USA; ^3^ Department of Engineering Science and Mechanics Pennsylvania State University University Park PA USA; ^4^ Nevada Seismological Laboratory University of Nevada Reno NV USA; ^5^ Department of Geosciences Pennsylvania State University University Park PA USA; ^6^ Dipartimento di Scienze della Terra La Sapienza Università di Roma Rome Italy

## Abstract

Tectonic faults fail through a spectrum of slip modes, ranging from slow aseismic creep to rapid slip during earthquakes. Understanding the seismic radiation emitted during these slip modes is key for advancing earthquake science and earthquake hazard assessment. In this work, we use laboratory friction experiments instrumented with ultrasonic sensors to document the seismic radiation properties of slow and fast laboratory earthquakes. Stick‐slip experiments were conducted at a constant loading rate of 8 μm/s and the normal stress was systematically increased from 7 to 15 MPa. We produced a full spectrum of slip modes by modulating the loading stiffness in tandem with the fault zone normal stress. Acoustic emission data were recorded continuously at 5 MHz. We demonstrate that the full continuum of slip modes radiate measurable high‐frequency energy between 100 and 500 kHz, including the slowest events that have peak fault slip rates <100 μm/s. The peak amplitude of the high‐frequency time‐domain signals scales systematically with fault slip velocity. Stable sliding experiments further support the connection between fault slip rate and high‐frequency radiation. Experiments demonstrate that the origin of the high‐frequency energy is fundamentally linked to changes in fault slip rate, shear strain, and breaking of contact junctions within the fault gouge. Our results suggest that having measurements close to the fault zone may be key for documenting seismic radiation properties and fully understanding the connection between different slip modes.

## Key Points

- We document seismic radiation properties of laboratory earthquakes for a spectrum of failure modes from stable sliding to fast stick‐slip
- The full spectrum of labquake failure modes radiate measurable high‐frequency energy between 100 and 500 kHz
- High‐frequency energy in slow and fast laboratory earthquakes scales systematically with fault slip rate

## 1 Introduction

Over the past 20+ years, broadband seismometers and continuous global positioning system (GPS) networks have combined to illuminate a continuum of fault slip behaviors. These observations show that tectonic faults store and release elastic‐strain energy through a spectrum of slip regimes ranging from slow, aseismic slip to fast, dynamic earthquake ruptures (e.g., Behr & Burgmann, 2020; Beroza & Ide, 2011; Peng & Gomberg 2010). Slow earthquakes encompass a range of slip modes that include aseismic creep, very‐low frequency earthquakes (VLFE), low‐frequency earthquakes (LFE), non‐volcanic tremor (NVT), and episodic tremor and slip (ETS) (Obara, 2002; Rogers & Dragert, 2003; Shelly et al., 2007). Since the discovery of slow earthquakes, a fundamental goal in earthquake seismology has been to elucidate the connections, and the possibility of fundamental differences, between slow and regular earthquakes (Obara & Kato, 2016). If the underlying physics associated with slow earthquakes is similar to that of ordinary earthquakes, then we can use observations of slow earthquakes to strengthen our understanding of the larger, fast earthquakes that pose the most exigent seismic hazard. Furthermore, it is thought that slow slip events can evolve into or trigger larger megathrust earthquakes (e.g., Segall & Bradley, 2012; Socquet et al., 2017), and thus, tracking the spatiotemporal properties of slow events could help advance seismic hazard analysis, earthquake early warning systems, and statistical forecasting (Kato et al., 2012; Obaro & Kato, 2016).

Waveforms from slow tectonic earthquakes are depleted in high‐frequency energy compared to ordinary earthquakes of equivalent size (Ito et al., 2007; Obara, 2002; Shelly et al., 2007). The amplitude spectra of tremor, LFEs, and VLFEs show that these events have low corner frequencies and are enriched in low‐frequency energy between 0.005 and 10 Hz (Kao et al., 2005; Rubinstein et al., 2007; Shelly et al., 2007). This depletion in high‐frequency energy can also be seen in the raw time‐domain signals (Kao et al., 2005; Shelly et al., 2007). Because slow earthquakes typically occur in frictionally transitional regions that bound the traditional seismogenic zone and may feature high in‐situ fluid pressures (Behr & Burgmann, 2020) or structural anisotropy (Miller et al., 2021), it is possible that variations in path effects, such as a low‐velocity zone within the source region could have a significant effect on the spectra of slow earthquakes (Bostock et al., 2017; Gomberg et al., 2012). Alternatively, the lack of high‐frequency energy in slow earthquakes could be linked to their source properties (Bostock et al., 2015; Thomas et al., 2016). For example, Thomas et al. (2016) pointed out that the depletion of high‐frequency energy in LFEs can be explained by slow slip rates, low stress drops, and slow rupture velocities.

Ultimately, the spectral properties of slow and fast events have significant implications for earthquake scaling laws, earthquake nucleation, and earthquake rupture. If the source properties of slow earthquakes are fundamentally different, then they should obey different scaling laws and their underlying physics could be different than regular earthquakes (Ide et al., 2007). However, the relationship between slow and fast ruptures is not well understood and it is clear that more work is needed to help establish the connection between slow and fast earthquakes (Frank & Brodsky, 2019; Michel et al., 2019; Supino et al., 2020)

In this work, we highlight seismic radiation properties of slow and fast laboratory earthquakes. We use a suite of laboratory friction experiments and data from ultrasonic transducers to elucidate the acoustic signatures of slow and fast stick‐slip events. The full spectrum of failure modes generate measurable high‐frequency energy between 100 and 500 kHz, including creep and slow laboratory earthquakes with peak fault slip rates <100 μm/s. The time‐domain peak amplitudes of the high‐frequency signals scale systematically with event size and are modulated by fault slip rate. These measurements are made possible by having high‐resolution seismic recordings located near the fault zone.

### 1.1 Unconfined and Confined Laboratory Earthquakes

In general, laboratory earthquakes can be subdivided into two main categories: (a) unconfined instabilities that rupture the entire fault plane and behave like a rigid 1D spring‐slider system and (b) confined instabilities that initiate from within the sample and terminate before rupturing the entire fault plane. To understand the physical significance of the work presented here, it is important to note that the laboratory stick‐slip events reported on in this study differ from other fault rupture experiments in the literature that involve meter‐long faults or compliant analogue materials (e.g., Ben‐David et al., 2010; Latour et al., 2013; Wu & McLaskey, 2019). In those experiments, the compliance of the loading apparatus is dominated by the sample stiffness, rather than the machine stiffness and laboratory earthquakes can nucleate from within the sample (see McLaskey & Yamashita, 2017). Such boundary conditions can promote confined events, where lab earthquakes nucleate, propagate, and terminate before reaching the ends of the sample. In contrast, for the stick‐slip events reported in this study, the compliance of the loading apparatus is dominated by the machine stiffness, and not the sample stiffness. Hence, the fault behaves like a rigid 1D spring‐slider system, producing system‐spanning and unconfined laboratory earthquakes. Parameters such as stress drop and seismic moment reported in this study are derived from unconfined stick‐slip events that rupture the entire 100 × 100 mm^2^ fault surface, and thus, may not be completely comparable to the measurements made in the fault rupture studies described above or the source spectral properties derived from seismic signals (Brune, 1970; Hanks & Wyss, 1972; Savage, 1972). Nevertheless, we report on high precision measurements that are made very close to the fault zone, which may offer new insights into the mechanics and seismic signatures of slow and fast laboratory earthquakes.

## 2 Acoustic Monitoring of Slow and Fast Laboratory Earthquakes

**Figure 1:**
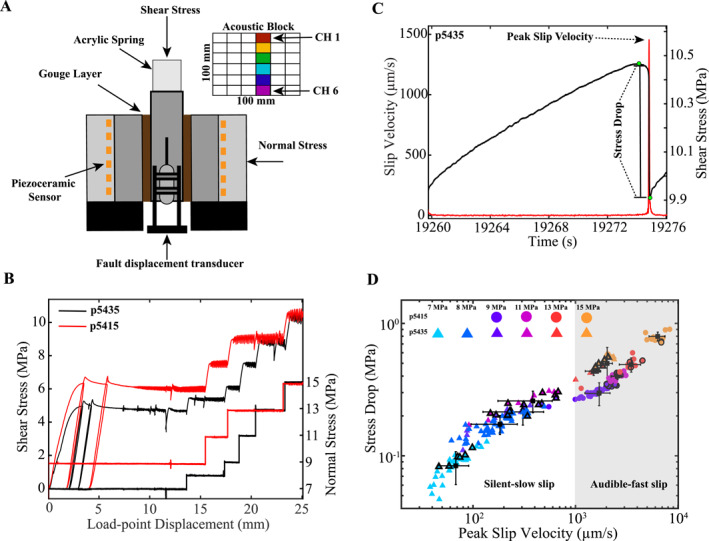
(a) Double‐direct shear (DDS) configuration showing two layers of fault gouge sandwiched between three steel forcing blocks. Fault displacement is measured between the center block and the base plate. Steel loading platens on each side of the DDS have piezoceramic transducers (orange squares) at the bottom of blind holes, 22 mm from the edge of the fault. Top inset shows AE channels. (b) Shear stress and normal stress plotted versus time for two experiments. Each experiment starts with a period of stable sliding, followed by the onset of slow stick‐slip after ∼10 mm of shear. (c) Slip velocity and shear stress evolution for one seismic cycle. For each slip cycle, we compute the stress drop and peak slip rate. Stress drop is computed as the difference between the maximum and minimum shear stress (green circles). (d) Stress drop as a function of peak slip velocity. Circles represent data from Experiment p5415 and triangles represent data from Experiment p5435; symbols are color coded according to normal stress. Symbols with thick black edges denote stick‐slip events that contain measured AE data. Black symbols represent averages at each normal stress and error bars represent one standard deviation. The transition between slow and fast stick‐slip occurs at ∼1 mm/s (see Leeman et al., 2016). Stress drop scales systematically with peak slip rate.

We performed a suite of friction experiments using a servo‐controlled biaxial deformation apparatus in a double‐direct shear (DDS) configuration (Figure 1a). We sheared 3 mm layers of quartz powder (median particle size 10.5 μm) over a range of normal stresses from 7 to 15 MPa at a fixed loading rate of 8 μm/s. Samples were constructed using steel side blocks of dimensions 100 × 100 × 20 mm and a steel center block of 100 × 150 × 30 mm. A constant frictional contact area (100 × 100 mm) was maintained throughout shear. The forcing blocks contain 0.8 mm deep grooves with a 1 mm wavelength to eliminate boundary shear. Fault displacements normal and parallel to the shear direction were measured by direct current displacement transformers (DCDT). Shear and normal loads were measured with strain gauge load cells mounted in series with the load axes. We measured fault slip with a DCDT spanning the bottom of the DDS center block and the base plate of the testing machine (Figure 1a). Fault displacements and stresses were measured continuously throughout the experiment at 100 Hz sampling rate. Experiments were conducted at 100% relative humidity by enclosing the DDS inside a plastic membrane and circulating humid air around the sample throughout the experiment (see Bolton et al., 2020).

We document a continuum of slip behaviors from stable sliding to fast stick‐slip events (i.e., laboratory earthquakes) with peak slip rates ranging from ∼50 μm/s to ∼5 mm/s (i.e., 625 *V*_L,_ where *V*_L_ is the far field loading rate) (Figure 1). We modulated the loading stiffness *k* to maintain near‐critical levels (i.e., *k/k*_*c*_ ∼ 1), and systematically change the fault normal stress to produce both slow and fast laboratory earthquakes, where *k*_*c*_ is the critical fault weakening rate (e.g., Leeman et al., 2016; Scuderi et al., 2016; 2017, Figure 1).

Acoustic emission (AE) data were measured continuously for 3–5 seismic cycles at each normal stress using broadband (∼0.0001–2 MHz) piezoceramic sensors (6.25 mm diameter; 4 mm thick), with a peak sensitivity between 100 and 500 kHz. The sensors are epoxied inside steel blocks and positioned ∼22 mm from the edge of the fault zone (Figure 1). Acoustic data were sampled continuously at 25 MHz and decimated to 5 MHz using a 16‐bit National Instruments PXIe‐5171 data acquisition system. To avoid clipping the co‐seismic AEs while maximizing signal‐to‐noise ratio, AE data were digitized over an adjustable vertical range spanning ±200–1400 mV. It should also be noted that the acoustic signals were not pre‐amplified/filtered during the data acquisition process, and thus only the (energetic) AE signals that exceed the background noise were recorded. Acoustic data were recorded from six channels oriented parallel to the direction of shear (see Figure 1 inset). Because the acoustic sensors are spaced closely together (16.8–84.0 mm), we observe negligible differences in the overall spectral properties of individual slip events recorded across our seismic network (Figure S1 in Supporting Information S1; Bolton et al., 2021; Rivière et al., 2018). Therefore, we present results derived from one channel (channel 1) and assume that our results are representative of all channels (see Figure S1 in Supporting Information S1). It is important to note that we record AE data for 3–5 slip cycles at each normal stress. Thus, we have far more geodetic measurements (e.g., Figure 1d) compared to seismic measurements.

## 3 Results

### 3.1 Source Properties of Slow and Fast Laboratory Earthquakes

**Figure 2:**
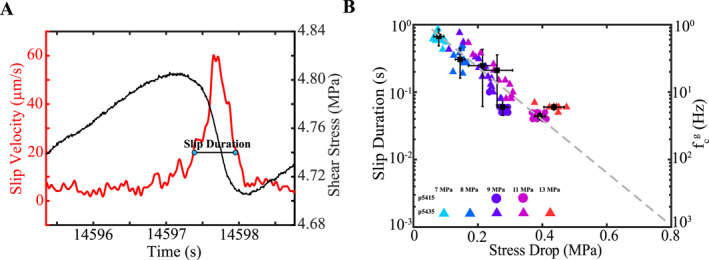
(a) Slip velocity and shear stress for a slow slip event. Blue symbols represent 20% of the peak slip rate. Slip duration is defined as the time difference between the two blue symbols. (b) Slip duration and geodetic corner frequency (f_c_^g^; 1/slip duration) scale inversely with stress drop. We do not include the fastest events at 13 and 15 due to the limited resolution we have from sampling at 100 Hz. Gray dotted line represents extrapolation of data down to the highest stress drops where we would expect a slip duration of 1 ms (f_c_^g^ = 1,000 Hz).

Each stick‐slip experiment was conducted at a constant loading rate of 8 μm/s and begins with a period of stable of sliding followed by the onset of slow (silent) laboratory earthquakes, and finally transitions into fast (audible) stick‐slip events (Figure 1). This progression occurs naturally (Leeman et al., 2016) and is hastened by increasing normal stress from 7 to 15 MPa (Figure 1b). We quantified the size of each stick‐slip event by measuring the stress drop (Figure 1c), peak fault slip rate, and slip duration (Figure 2). Stress drop is measured as the difference between the peak shear stress and minimum shear stress of the co‐seismic slip phase (Figure 1c). The peak fault slip rate is measured from the time derivative of the fault displacement as measured by the local transducer on the DDS center block (see Figure 1a). Note that this measurement represents the average fault motion and differs from the far‐field loading rate, which is held constant. In addition, the peak slip velocities reported in this study represent a spatially averaged slip rate and is possible that discrete patches on the fault experience higher local slip rates.

We find that stress drop and peak slip rate scale systematically with fault normal stress, consistent with previous studies (Figure 1d; Leeman et al., 2016; Passelègue et al., 2016; Passelègue et al., 2020; Tinti et al., 2016; Wu & McLaskey, 2019). We classify slip events as slow or fast based on their peak slip velocities: slip events with peak slip velocities <1 mm/s (i.e., <125 *V*_L_) are defined as slow and those with peak slip rates ≥1 mm/s are fast. This threshold marks the transition from silent to audible stick‐slip and is consistent with definitions used in previous studies (e.g., Leeman et al., 2016; Shreedharan et al., 2020).

After correcting for path and site effects, the displacement spectrum of a circular rupture has a corner frequency that is inversely related to earthquake duration, T: *f*_c_ ∼ 1/T. However, because path and site effects have not been decoupled from our seismic recordings we are unable to estimate a corner frequency from the seismic spectra. Instead we derive a “geodetic” based corner frequency (*f*_c_^g^) from the slip duration recorded by the fault displacement sensor located on the center block of the DDS (Figure 2). We estimate the slip duration and *f*_c_^g^ from the slip velocity because its definition is directly associated with the phase of rapid fault acceleration and deceleration that most effectively radiates seismic waves. In particular, we identify when the slip rate exceeds 20% of its peak during the acceleration and deceleration phase and take the difference between these two time stamps as a measure of the slip duration (Figure 2a). The 20% threshold is arbitrary, but this produces slip durations that are consistent with previous laboratory studies (Leeman et al., 2016; Shreedharan et al., 2020; Figure 2b). However, because mechanical data are recorded at 100 Hz we have limited temporal resolution for slip events at 13 and 15 MPa. To estimate the slip duration for these events, we extrapolate the trend in Figure 2b down to the highest stress drops. This extrapolation indicates that fast laboratory events have slip durations of ∼1 ms (i.e., *f*_c_^g^ = 1 kHz). Slow laboratory events have slip durations between 0.1 and 1s and *f*_c_^g^ between 1 and 10 Hz. From here forward we refer to low‐frequency signals as *f* ≤ *f*_c_^g^ and high‐frequency signals as *f* > *f*_c_^g^.

Our *f*_c_^g^ is not completely analogous to the standard corner frequency *f*_c_ estimated in seismic source studies (Aki, 1967; Brune, 1970; Hanks & Wyss, 1972; Ide et al., 2007) which is often related to the radius of the ruptured fault area, assuming a confined‐circular rupture with radius *r* and constant rupture velocity of order 90% of the shear wave speed *V*_*s*_:

(1) $$f_{c}∼\frac{0.9*V_{s}}{r}\;$$

The stick‐slip events reported in this study are unconfined and do not have a measurable rupture velocity. In addition, event durations appear to be primarily related to their rise time (i.e., the time that it takes for the fault to reach its maximum slip displacement at a particular location on the fault), rather than then overall rupture dimension. Therefore, the slip duration and the geodetic corner frequency that we derive in Figure 2 should not be used to estimate the spatial size of the source, demark slow and fast stick‐slip events, or compared to values that would be obtained by using Equation 1 above. However, differences between *f*_c_^g^ and *f*_c_ have little effect on the results of this work since we do not attempt to interpret the physical significance of *f*_c_^g^, and only use it as a reference point to differentiate between low and high‐frequency signals.

### 3.2 Spectral Characteristics of Slow and Fast Laboratory Earthquakes

The raw continuous AE signals we report are not filtered, and thus, are contaminated with low‐frequency (<10 kHz) electrical and mechanical noise (Figure S2 in Supporting Information S1). We do not filter the signals during data acquisition because we are interested in preserving the full bandwidth of the seismic signals. Unfortunately, the hydraulic power‐supply (HPS) adds significant noise to the acoustic signals at low frequencies (Figures S1–S3 in Supporting Information S1). The effect of the HPS can be seen clearly by comparing the time series and spectra of recorded AE signals with and without the HPS turned on (Figure S2 in Supporting Information S1). The HPS radiates energy between ∼0 and 10,000 Hz and has significant impact on frequencies <1,000 Hz, where it adds ∼2 orders of magnitude in noise. It is also important to note that the HPS noise is in the same bandwidth as the slip events (see Figure S3 in Supporting Information S1), thus the HPS noise cannot be removed with standard filtering approaches. The HPS noise hinders our ability to comment on the spectral characteristics in Figure S3 in Supporting Information S1 because the signal‐to‐noise‐ratio (SNR) is low, particularly at low frequencies. If we follow the convention of previous laboratory studies (Wu & McLaskey, 2019), then a SNR ≥ 20 dB is needed to confidently assess the data in Figure S3 in Supporting Information S1. Because this is not feasible we focus our analysis on the time‐domain characteristics at the higher frequencies (>10 kHz) that are not contaminated by HPS noise.

**Figure 3:**
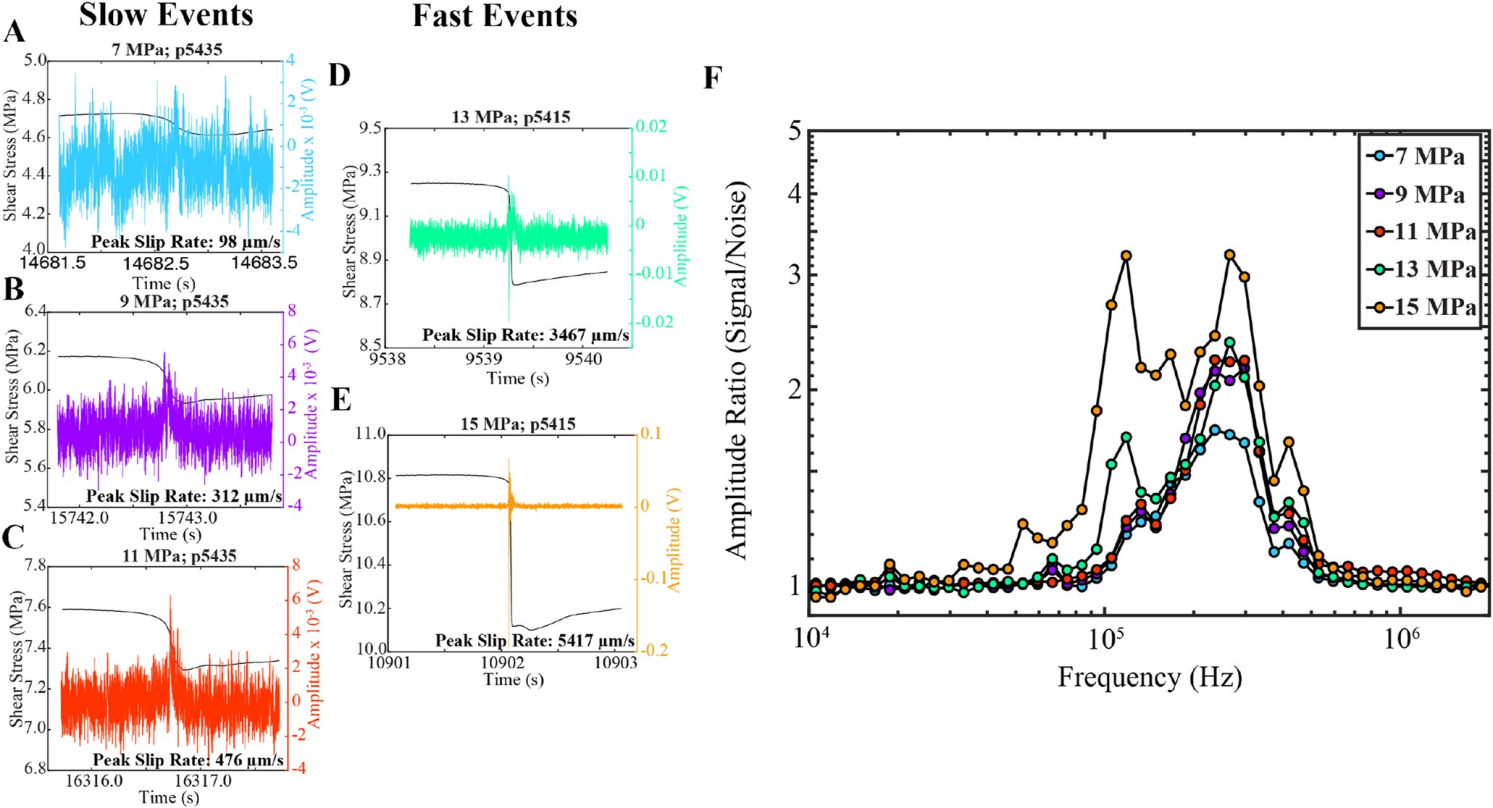
(a)–(e) Shear stress and AE amplitude evolution during co‐seismic slip for a representative series of slow to fast laboratory earthquakes, with peak slip rates spanning between 98 and 5,417 μm/s. Acoustic traces are 2s long and correspond to Channel 1. (f) Signal to noise ratios derived from spectra in Figure S3. Slow events (7–11 MPa) have poor SNR across most of their bandwidth, with values slightly higher than 1 within the 100–500 kHz bandwidth. Fast events (13–15 MPa) have higher SNR for frequencies <10 kHz and between 80 and 500 kHz.

AE time series and shear stress evolution for a representative set of slow and fast events are illustrated in Figures 3a–3e. Acoustic traces are 2 s long and centered about their peak AE amplitude reached during co‐seismic slip. We plot spectral ratios of signal‐to‐noise between 0.01 and 2 MHz in Figure 3f. Although our acoustic sensors are not absolutely calibrated, we suspect that the sensor response is a function of frequency and is proportional to displacement for frequencies between 100 and 800 kHz (Wu & McLaskey, 2018). Prior to computing the spectra we remove the mean and taper the acoustic traces with a Kaiser window. Noise spectra are derived from 2 s long traces that occur during the initial stages of the seismic cycle (Figure S3a inset in Supporting Information S1). The initial stage of the seismic cycle is associated with low levels of AE activity, and thus, represents an ideal location to quantify the noise (see Bolton et al., 2020). Furthermore, because the acoustic signals are not pre‐amplified, only the co‐seismic slip events are detected (i.e., pre‐seismic AEs are below the noise level). Following Wu and McLaskey (2018), we perform a logarithmic averaging scheme in the frequency domain after computing the discrete Fourier transform (DFT). This averaging procedure ensures that the frequency bins are equally spaced on a log scale (∼20 bins per decade in frequency) and requires at least three samples of the DFT for each frequency bin. As a result of our 2s long time windows and the averaging scheme, our amplitude spectra plots span from ∼11 Hz to 2.5 MHz.

We observe a high‐frequency band between ∼100 and 500 kHz with elevated spectral amplitudes, irrespective of the size of the slip event (Figure 3f; Figure S3 in Supporting Information S1). The center frequency of the acoustic sensors is between ∼100 and 500 kHz, making the excitation of radiated energy most readily observed in this band. Recall that our definition of high‐frequency energy corresponds to frequencies larger than the geodetic corner frequency (Figure 2b). The slow events measured in this study have an *f*_c_^g^ between 1 and 10 Hz and have a high‐frequency component that is ∼4–5 orders of magnitude larger than *f*_c_^g^ (Figure 3f).

The high‐frequency content of slow laboratory earthquakes (i.e., normal stress of 7–11 MPa in Figures 3a–3c) is modestly different within the 100–400 kHz bandwidth, with faster events radiating more high‐frequency energy (Figure 3f). It is interesting to note that the slow events still radiate measurable high‐frequency energy between ∼100 and 500 kHz, despite having peak slip rates ≤100 μm/s. The spectral ratios of signal‐to‐noise for slow events increase from ∼100 kHz and reach a peak between 200 and 300 kHz (Figure 3f). As mentioned above, the data reported in Figure 3 are derived from one channel (channel 1) and one stick‐slip event from each normal stress. However, the characteristics in Figure 3 are generally representative of other channels/stick‐slip events (Figure S1 in Supporting Information S1).

Fast laboratory earthquakes (13–15 MPa) radiate more high‐frequency energy between 100 and 500 kHz and in general have larger SNR across most of their bandwidth (Figure 3f; Figure S3 in Supporting Information S1). We expect that the ‘geodetic’ corner frequency of the fast events is ∼1,000 Hz based on extrapolation in Figure 2b. Thus, for the fast events, the high frequency waves we detected are about ∼2 orders of magnitude larger than their geodetic corner frequency (Figure 3f). The two dominant peaks around 100 and 300 kHz are likely due to resonance of the sensor.

### 3.3 Time‐Domain and High‐Frequency Characteristics

**Figure 4:**
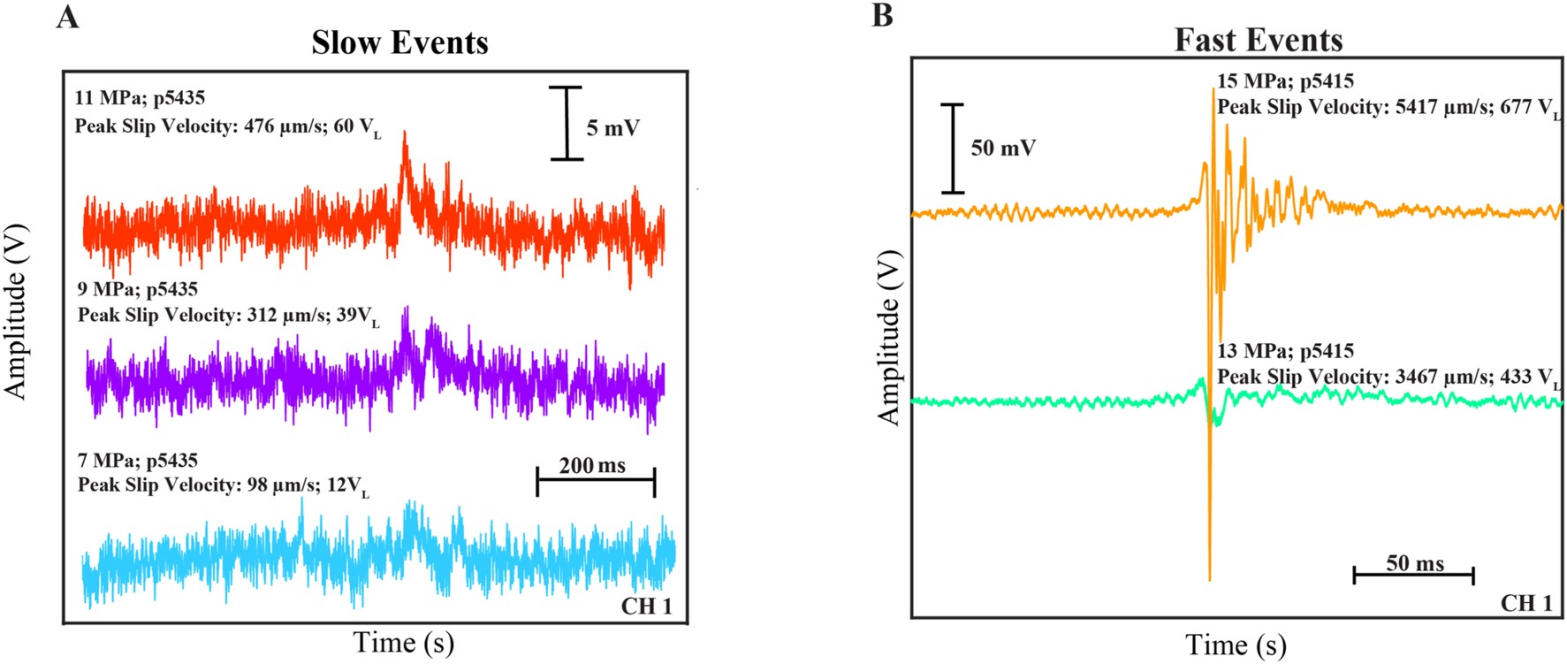
(a) Zoom of slow slip events from 7 to 11 MPa. Note that acoustic traces correspond to the same data plotted in Figure 2. Acoustic traces are centered about their peak amplitude and offset vertically for clarity. The time‐domain characteristics change slightly with normal stress and have a simplistic structure compared to the fast events in panel (b) (b) Zoom of time‐domain signatures of fast slip events at 13 and 15 MPa (same events in Figure 3). Both events are larger and more impulsive compared to the slow events in panel (a).

To distinguish the time‐domain signatures of slow and fast events, we plot snapshots of the AE signals associated with the co‐seismic stress drops (Figure 4). Peak slip rates for these events range between 98 and 5,417 μm/s, and thus represent a continuum of failure modes that are analogous to tectonic fault systems. The time‐domain signals in Figure 4 are similar to those reported in previous studies (e.g., Wu & McLaskey, 2019). In particular, slow laboratory events have broad‐time domain signals with low amplitudes that are slightly above the noise level (Figure 4a). The character of the main slip event (approximately in the center of the traces) is very simplistic and resembles an ideal source‐time function. On the other hand, the fast events (e.g., 15 MPa) have impulsive time‐domain signals, large amplitudes, and contain a significant degree of coda energy following the first arrival (Figure 4b).

**Figure 5:**
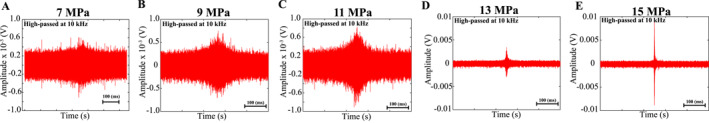
Signals from Figure 4 high‐pass filtered at 10 kHz. Both slow (a)–(c) and fast (d)–(e) slip events radiate high‐frequency energy. The high‐frequency pulse increases in size as events become progressively faster. Note the scale difference for fast events.

To illuminate the similarities and differences between slow and fast laboratory earthquakes, we high‐pass filter the acoustic traces (i.e., Figure 3) at 10 kHz with an eighth‐order IIR filter (Figure 5). We use a 10‐kHz cutoff frequency because frequencies below this are contaminated by the HPS noise (see Section 3.2 and Figure S2 in Supporting Information S1). Interestingly, all of the slip events analyzed contain remnants of high‐frequency energy (>10 kHz), consistent with the spectra in Figure 3f and Figure S3 in Supporting Information S1. Note, this high‐frequency energy exists even in the slowest events that have peak slip rates <100 μm/s and stress drops ∼100 kPa (e.g., Figure 5a). But note also sthat the high‐frequency pulse is narrow and impulsive for fast slip events whereas it is more diffuse and of lower amplitude for the slow events.

**Figure 6:**
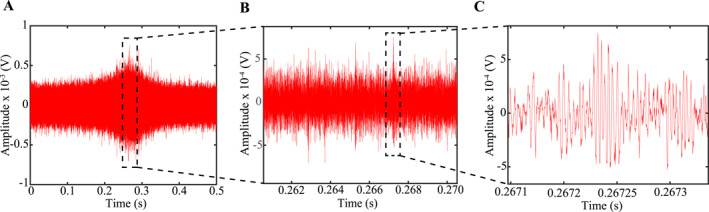
(a) High‐pass filtered signal from Figure 5B. (b) Zoom of the black rectangle box highlighted in panel (a) Note, the continuous signal is composed of several large/impulsive signals between 0.262 and 0.268 s. (c) Zoom of the black box highlighted in panel (b) At this scale it appears that the continuous signal is composed of several discrete AEs that are likely overlapping in time.

The high‐passed signals in Figure 5 look like a continuous burst of seismic energy, but careful inspection of the time‐domain signals reveals that this may not be the case (Figure 6). In Figure 6, we plot zoomed‐in snapshots from the high‐frequency pulse in Figure 5b. After zooming into the main pulse, it is clear that the continuous‐like signal is actually composed of a myriad of impulsive AEs (Figure 6b). These are the large and narrow spikes that appear in Figure 6b. We highlight one of these AEs with the black dotted box and plot a zoomed‐in version of the event in Figure 6c. At the microsecond scale it is unclear if the event in Figure 5b represents a single or multiple AE(s). The AEs in Figure 6c do not have clear phase‐arrivals, but broadly speaking they represent tiny packages of energy that are characteristic of AE signals (Figure 6c). The incoherency between individual AEs could be due to multiple small AEs that may be occurring quasi‐continuously, and thus, mask the details of any individual AE. In any case, it is clear that the high‐frequency pulses in Figure 5 are composed of multiple discrete AEs that occur roughly simultaneously throughout co‐seismic failure. It's also worth noting that the acoustic signals depicted in Figure 6 are remarkably similar to those found in previous laboratory stick‐slip experiments on PMMA and salt (e.g., McLaskey & Glasser, 2011; Zigone et al., 2011). McLaskey and Glasser (2011) were able to locate the AEs and interpreted them as localized slip events that ruptured micro‐scale asperities.

**Figure 7:**
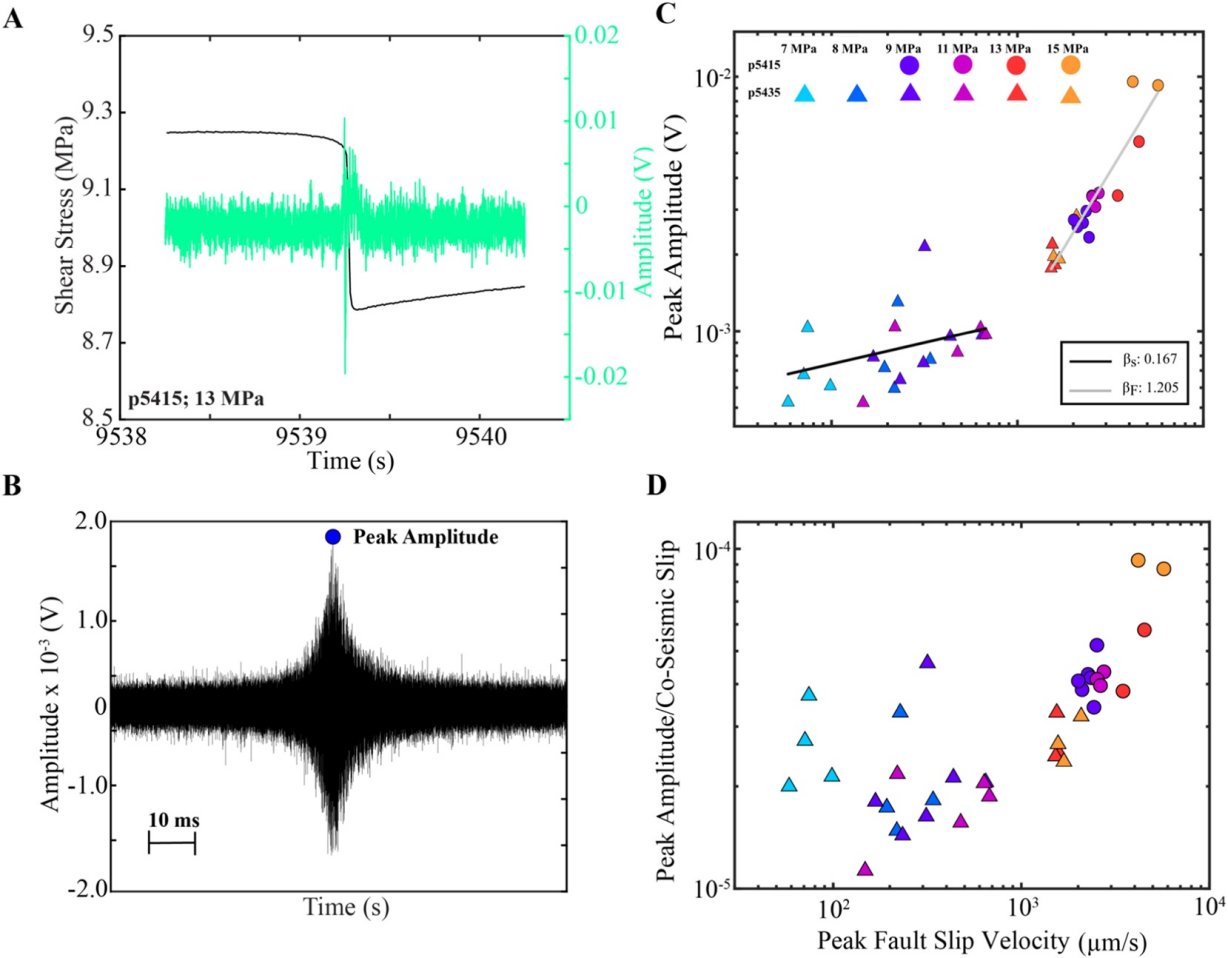
(a) Shear stress and AE time‐series for a fast event at 13 MPa. (b) AE signal from A after high‐pass filtering the signal at 10 kHz. We quantify the high‐frequency pulse my measuring its peak amplitude. (c) Peak amplitude of high‐frequency pulses (e.g., panel (b) as a function of fault slip velocity. *Note.* color scheme and symbols are the same as those depicted in Figure 1. Slow and fast events follow different scaling relationships as noted by differences in their slopes (β_s_ for slow and β_F_ for fast events). (d) Peak amplitudes of high‐frequency pulses scaled by co‐seismic slip. Scaled amplitudes for slow events are roughly independent of fault slip rate, while they increase systematically with fault slip rate for fast events.

To further assess the robustness of high frequency seismic radiation during slow slip, we measure the peak amplitude of the high‐passed acoustic signals in Figure 5 (Figure 7). Figure 7 shows such data for all stick‐slip events for which AE data are recorded. Recall, that AE data are only collected for a subset of the stick‐slip events in Figure 1d. The instrument response and path effects have not been decoupled from the data in Figure 7. However, we argue that such corrections will have modest effects on the relative changes in Figure 7 because the instrument response is approximately constant throughout the experiment, the bandwidth of the measurements is relatively narrow, and the acoustic waves traverse the same area (McLaskey et al., 2015).

This analysis demonstrates that the characteristics depicted in Figure 5 are common features among all of our stick‐slip events (Figure 7). The amplitudes of the high‐frequency time‐domain pulses scale systematically with fault slip velocity (Figure 7c). Two distinct clusters form in Figure 7c and are clearly differentiated as a function of slip velocity; events that fall below 1 mm/s are slow slip events, while those that plot above 1 mm/s are fast events. We perform a log‐log fit on these two clusters of data and denote their slopes by β_S_ (slow slip) and β_F_ (fast slip), respectively. Slow events have smaller slopes (β_S_) compared to faster events (β_F_) and the data show a notable break in slope between β_S_ and β_F_ at 1 mm/s. This break in slope is also consistent with the bifurcation separating the slow and fast slip events in Figure 1d (see also Shreedharan et al., 2020).

The scaling in Figure 7c is not surprising because larger stick‐slip events should radiate bigger and more energetic AEs due to a systematic increase in elastic‐strain energy at higher normal stresses. In our experiments, we observe system‐spanning slip events where the entire 100 × 100 mm^2^ fault area slips during failure. Because of this, changes in seismic moment are primarily governed by variations in co‐seismic slip (Figure S4 in Supporting Information S1). In our experiments, co‐seismic slip and seismic moment increase systematically with normal stress (Figure S4 in Supporting Information S1). The seismic moment that we report in Figure S4 in Supporting Information S1 represents a ‘geodetic moment’ and thus may not be directly comparable to a seismic moment measured from the low‐frequency portion of a seismogram since the two measurement modalities are fundamentally different. To account for differences in stick‐slip magnitude we normalize the high‐frequency peak amplitudes in Figure 7c by co‐seismic slip (Figure 7d). The normalized amplitudes for the slow events are roughly independent of peak slip rate, while for the fast events scale systematically with fault slip rate.

## 4 Discussion

Seismic waves contain information about earthquake source properties and are key for establishing scaling laws of earthquake rupture (Aki, 1967; Brune, 1970; Ide & Beroza, 2001). If the physical processes that govern earthquake rupture are invariant to earthquake size, then earthquakes of all sizes should follow similar scaling laws (Aki, 1967; Prieto et al., 2004). However, slow earthquake families (SSE, LFE, VLFE, tectonic tremor) are something of a conundrum in earthquake science because they are depleted in high‐frequency energy (>10 Hz) and have low corner frequencies, slip rates, stress drops, and rupture velocities when compared to regular earthquakes of equivalent size. These differences have been used to suggest that slow and fast events obey different scaling laws (e.g., moment‐duration), which in turn could reflect fundamental differences in their underlying physics (Bostock et al., 2015; Ide et al., 2007; Thomas et al., 2016).

However, recent work suggests that slow earthquakes may follow similar scaling relationships to regular earthquakes (Frank & Brodsky, 2019; Michel et al., 2019). In addition, laboratory studies show that a full continuum of slip modes, ranging from aseismic slip to fast‐dynamic ruptures can originate along the same fault due to changes in effective stiffness and frictional and fault zone properties (Aben & Brantut, 2021; Ben‐David et al., 2010; Bolton et al., 2020; Kaproth & Marone, 2013; Leeman et al., 2016; 2018; McLaskey & Yamashita, 2017; Passelègue et al., 2020; Scuderi et al., 2016; Shreedharan et al., 2020; 2017; 2020; Tinti et al., 2016; Wu & McLaskey, 2019). Here, we use results from unconfined laboratory stick‐slip experiments instrumented with ultrasonic transducers and illuminate the seismic radiation properties of slow and fast laboratory earthquakes.

### 4.1 The High‐Frequency Signature of Slow and Fast Laboratory Earthquakes

We document systematic differences and similarities in the time‐domain signatures of slow and fast laboratory earthquakes. We high‐pass filter the time‐domain signals at 10 kHz and demonstrate that both slow and fast laboratory earthquakes emanate high‐frequency energy (Figures 5, 6, 7). Our data are in good agreement with previous laboratory studies that measure the high‐frequency energy recorded during fast slip events (McLaskey & Glaser, 2011; McLaskey et al., 2012; Marty et al., 2019; Wu and McLaskey, 2019). In addition, we show that the peak amplitudes of the high‐frequency pulses increase systematically with fault slip velocity, with faster slip events associated with more energetic events (Figure 7). This is consistent with seismic observations showing that tectonic earthquakes with large stress drops radiate more high‐frequency energy (e.g., Boore, 1983; Hanks & McGuire, 1981; Oth et al., 2017; Trugman & Shearer, 2018). We follow the interpretation of previous works and propose that earthquakes with large stress drops and high slip velocities radiate more high‐frequency energy due to a systematic increase in frictional healing during the inter‐seismic period (McLaskey et al., 2012). Enhanced frictional healing at higher normal loads is supported by data in Figure S4 in Supporting Information S1, showing that peak friction, seismic moment, and recurrence interval all increase with normal stress (see also Karner & Marone, 2000). Hence, frictional healing produces larger and faster laboratory earthquakes at higher normal stresses, which in turn, radiate larger amounts of high‐frequency energy.

The data in Figure 7c show that the high‐frequency amplitudes of the time‐domain pulses increase by a factor of ∼50, while the slip rate changes by two orders of magnitude. This observation could point to fundamental differences between the geodetic and seismic signatures of source processes in laboratory earthquakes. However, because our acoustic sensors are not absolutely calibrated it remains difficult to draw conclusions about the absolute values between the two measurements. On the other hand, it is also plausible that the seismic signature of the source is much weaker than the geodetic signature simply due to attenuation.

The data in Figures 7c and 7d indicate that the high‐frequency characteristics of slow and fast stick‐slip events may follow different scaling relationships. To account for differences in event size, we normalize the peak amplitudes by the total co‐seismic slip during each stick‐slip event. This effectively normalizes the high‐frequency amplitudes by stick‐slip magnitude and is in some ways analogous to a measure of scaled energy where the radiated energy is normalized by seismic moment (e.g., Ide & Beroza, 2001). For slow events, the scaled amplitudes are roughly independent of fault slip rate. In contrast, for fast events the scaled high‐frequency amplitudes increase with fault slip velocity, suggesting that faster events are more efficient at radiating high‐frequency energy compared to slow events. This implies that modest changes in frictional properties and boundary conditions along a single fault patch can produce significant changes in seismic radiation properties (e.g., Veedu & Barbot, 2016). In addition, the data in Figure 7 indicate that fast stick‐slip events radiate more energy per unit increase in fault slip compared to slow events. Hence, the seismic properties of slow and fast events maybe fundamentally different, which is consistent with the scaling laws identified in previous laboratory studies (Wu & McLaskey, 2019). On the other hand, it's possible that the break in scaling in Figure 7 is due to a frequency shift from the response of the AE sensors. However, we are argue that this is unlikely because: 1.) the break in scaling also occurs in our fault slip measurements (Figures 1d and 2) most of the high‐frequency energy lies between 80 and 500 kHz and we do not expect the response of the sensors to vary over such a narrow bandwidth (see Wu & McLaskey, 2018).

In contrast to previous laboratory studies, our data show that slow laboratory earthquakes with peak slip rates <100 μm/s and stress drops <100 kPa radiate detectable high‐frequency energy (>10 kHz) (Figure 5; McLaskey & Yamashita, 2017; Wu & McLaskey, 2019). We propose that attenuation plays a key role in the ability to measure high‐frequency radiation during slow and fast slip events. The geometry of our setup along with our high‐resolution measurements allow us to measure small amounts of high‐frequency energy generated during slow slip (Figure 1a). More specifically, our acoustic sensors are placed ∼22 mm from the edge of the fault and the acoustic waves that are radiated during co‐seismic failure spend most of their time propagating through the steel loading blocks (Figure 1a). Aside from the thin gouge layer where the events nucleate, the acoustic waves do not propagate through a highly attenuating medium, such as a low‐velocity zone, where the loss of high‐frequency seismic waves is more likely to occur (e.g., Bostock et al., 2017; Gomberg et al., 2012). Furthermore, in our laboratory experiments the entire 100 × 100 mm^2^ fault patch slips during co‐seismic failure and our sensors are only 22 mm away from the source. This fact could help explain why previous laboratory studies were unable to measure high‐frequency energy in slow laboratory earthquakes. For example, the slowest events reported in Wu and Mclaskey (2019) have peak slip rates of 500 μm/s (5× faster than our slowest events), but lack a detectable high‐frequency signature. However, their sensors are located ∼200 mm from the edge of the fault and the radiated waves propagated through a thick block of granite (as opposed to the steel loading blocks in our case). It is possible that the slow events reported in Wu and McLaskey (2019) did in fact radiate high‐frequency energy but the high‐frequency waves were significantly attenuated before reaching the sensors. The high‐frequency signature of slow tectonic earthquakes may be difficult to observe for a similar reason.

Our work implies that the source‐sensor geometry and the medium surrounding the fault zone could have a significant effect on high‐frequency characteristics. This highlights the importance of having measurements close to the fault zone and suggests that careful corrections for near‐source attenuation is essential in the accurate measurement of earthquake source properties (e.g., Abercrombie et al., 2021).

### 4.2 The Causal Processes That Regulate High‐Frequency Energy in Laboratory Earthquakes

The data presented in Figures 3, 4, 5, 6, 7 suggest that slow laboratory earthquakes radiate high‐frequency energy. Given that both slow and fast laboratory earthquakes radiate high‐frequency energy, it is plausible that the physical mechanism responsible for the high‐frequency energy is common to both of styles of rupture. The systematic scaling in Figures 7c and 7d seems to indicate that the high‐frequency energy is regulated by fault slip velocity. However, it is also possible that the data in Figure 7 are controlled by other processes, such as shear localization.

Shear localization is a common feature in tectonic faulting and plays a key role in modulating frictional behavior (Bedford & Faulkner, 2021; Kenigsberg et al., 2019; 2020; Marone & Kilgore, 1993; Marone, 1998; Marone et al., 1990; Mair et al., 2002; Mair & Marone, 1999; McBeck et al., 2018; 2021; Renard et al., 2019; Scuderi et al., 2017). Moreover, it has been suggested that shear localization plays a key role regulating seismic activity at the laboratory and tectonic scale (Ben‐Zion & Zaliapin, 2020; Dresen et al., 2020; Goebel et al., 2017; Lockner et al., 1991). Therefore, it is conceivable that the high‐frequency radiation we measure in our experiments is influenced by shear localization and shear strain effects.

**Figure 8:**
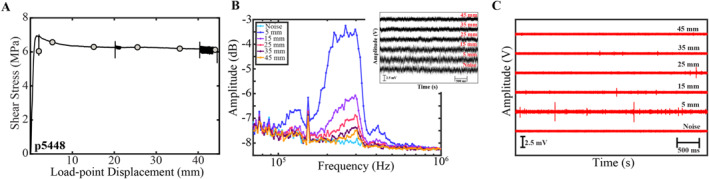
(a) Shear stress as a function of load‐point displacement for a stable sliding experiment. Gray symbols denote time stamps of where AE data are derived in panels (b) and (c) (b) Amplitude spectra for 2 s time series traces shown in top inset. Noise trace was derived from a hold during the beginning of the experiment (see panel A). AE signals have measurable frequencies between 100 and 500 kHz, consistent with data in the main text. Spectral amplitudes are highest after shearing 5 mm and subsequently decrease with increasing slip displacement. (c) Acoustic traces from panel B high‐passed filtered between 150 and 400 kHz. Spikes represent high‐frequency acoustic emissions. Consistent with panel (b), data show that AE activity is highest during the first 5 mm of shear and decrease subsequently with slip.

We address the role of shear localization by conducting a stable‐sliding experiment and measuring acoustic emission data as a function of slip displacement (Figure 8). We permit stable‐sliding by increasing the loading stiffness, *k,* which generates stable creep and eliminates stick‐slip instabilities (Leeman et al., 2016). In this experiment, the normal stress and shear velocity are held constant, allowing us to directly connect changes in high‐frequency radiation to cumulative slip displacement and shear localization (Scuderi et al., 2017). Similar to data above we compute the amplitude spectra of 2 s long acoustic traces derived from channel 1. Data show that the high‐frequency energy between 100 and 500 kHz scales inversely with shear displacement (Figure 8). We high‐pass filter the data between 150 and 400 kHz and demonstrate that acoustic emission activity is enhanced during the early stages of the experiment and decays non‐linearly with slip displacement. This indicates that high‐frequency radiation is enhanced when shear is more pervasive/distributed and decreases as shear becomes more localized. We thus argue that while shear localization may play an important role in modulating seismic characteristics, it cannot fully explain the data presented in Figures 3, 4, 5, 6, 7 (see also Shreedharan et al., 2020).

**Figure 9:**
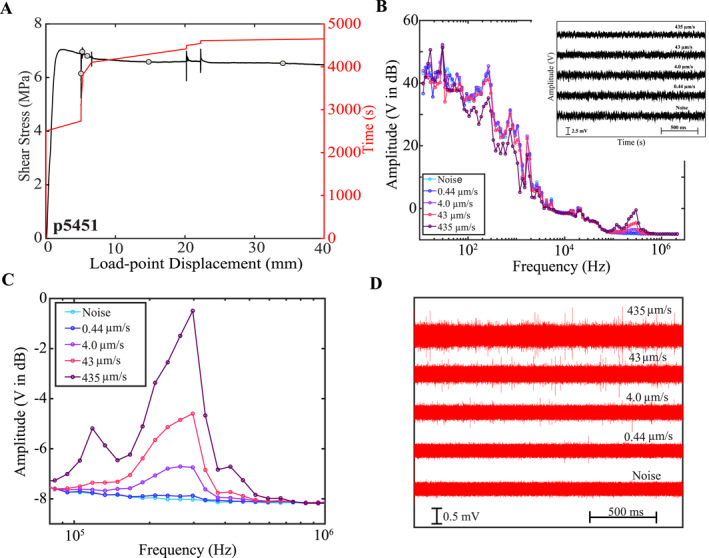
(a) Shear stress and time versus load‐point displacement for a stable sliding experiment. Loading‐rate was increased from 0.44 μm/s to 435 μm/s. Gray circles represent the time stamps from which AE traces are derived in panel (b) (b) Amplitude spectra derived from the 2s long acoustic traces (see inset) for each load‐point‐velocity. The noise trace was collected during a hold at the beginning of the experiment (see panel A). (c) Zoom of high‐frequency components in (b) The amplitudes and bandwidth of the high‐frequency energy increase with loading‐rate. (d) Acoustic traces from B band‐passed between 150 and 400 kHz. The size and number of AEs increases with loading rate.

Based on previous works we know that AE properties are strongly affected by changes in inelastic creep and fault slip rate (Bolton et al., 2020; 2021; Dresen et al., 2020; Fan et al., 2010; McLaskey & Lockner, 2014; Zigone et al., 2011). Thus, we propose that the origin of high‐frequency energy in our stick‐slip experiments is connected to fault slip rate and the breaking/sliding of grain contact junctions (e.g., McLaskey & Glaser, 2011). To test this idea, we conducted a stable‐sliding experiment at a constant normal load of 9 MPa and swept through 4‐orders of magnitude in sliding velocity (Figure 9). We compute the amplitude spectra of 2 s long time traces for each shearing velocity (Figure 9b). Similar to the stick‐slip events, these data show a distinct high‐frequency band between 100 and 500 kHz that likely corresponds to the peak sensitivity of the sensor near its resonant frequency. The amplitude of this high frequency energy increases with shearing rate/fault slip rate (Figures 9b and 9c). We also band‐pass filter the acoustic traces in Figure 9b between 150 and 400 kHz and demonstrate that there is enhanced acoustic emission activity at higher slip rates (Figure 9d). Interestingly, the spectra for the stable sliding data at 4.0 and 43 μm/s slip rates are remarkably similar to the spectra of slow stick‐slip events.

Fracture mechanics models and laboratory data suggest that high‐frequency ground motion originates at the rupture front and is caused by abrupt acceleration and deceleration (Madariaga, 1977; 1983; Marty et al., 2019; Scholz, 2019). Similarly, it is likely that high‐frequency energy radiated from laboratory earthquakes and AE events originates from the breaking and sliding of grain contact junctions (Bolton et al., 2020; Scholz, 1968). If true, this mechanism could have important implications and connections to the elastic impact model proposed by Tsai and Hirth (2020). In the Tsai and Hirth model, the high‐frequency energy is modulated by elastic collisions within the fault gouge and can occur at any time during the rupture process (Tsai & Hirth, 2020; Tsai et al., 2021). This view is consistent with our stable sliding data, which shows that high‐frequency energy is radiated at all times during frictional sliding (Figures 8 and 9). Also similar to our experiments, the recorded spectrum of the model of Tsai and Hirth (2020) is generated by the superposition of frictionally mediated slip along a localized fault surface at low frequencies and the effects of elastic impacts and collisions within the fault gouge at high frequencies.

**Figure 10:**
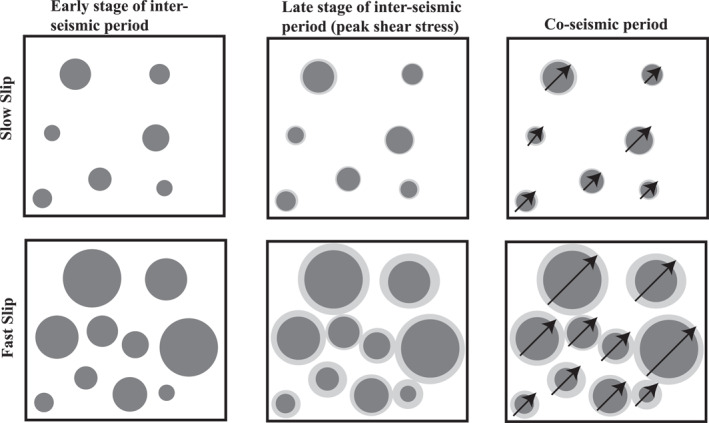
Schematic showing differences in micro‐mechanical processes for slow and fast ruptures. Circles depict 2D grain contact junctions; darker shades of gray represent contacts during the early stages of the inter‐seismic period while lighter shades of gray indicate contact growth via frictional healing and are representative of contacts prior to co‐seismic failure. Arrows during co‐seismic failure represent failure of contact junctions. Slow events experience less frictional healing during the inter‐seismic period, rupture smaller contacts, and radiate less acoustic energy. In contrast, fast events undergo more frictional healing during the inter‐seismic period, rupture larger contacts, and radiate more acoustic energy.

If acoustic emissions and laboratory seismicity are in fact broadband signals then our data indicate that high‐frequency radiation between 100 and 500 kHz is present in all slip modes ranging from creep to fast dynamic events in laboratory instabilities. We demonstrate that the high‐frequency energy radiated during these slip modes is modulated by fault slip rate and originates from the breaking and sliding of grain contact junctions (Bolton et al., 2020; McLaskey and Glaser, 2011; Zigone et al., 2011). Previous works demonstrate that AEs represent the failure of ∼mm size fault patches (Blanke et al., 2021; Goodfellow & Young, 2014; McLaskey & Lockner, 2014). If we follow this interpretation, then this implies that the discrete AEs that occur quasi‐continuously during the co‐seismic slip phase represent the failure of an ensemble of tiny fault patches (Figure 6). Hence, it's plausible that when the entire 100 × 100 mm^2^ fault plane slips during co‐seismic failure, multiple AEs broadcast energy from all across the fault plane. At higher normal stresses these fault patches experience more frictional healing during the inter‐seismic period, and this causes larger strength loss, larger stress drops, and faster slip rates that promote more high‐frequency seismic radiation during the co‐seismic slip phase (Figure 10; Figure S4 in Supporting Information S1). This view is also consistent with field observations that demonstrate that extended tectonic tremor is actually composed of individual low‐frequency earthquakes (e.g., Shelly et al., 2007). Our observations are also in good agreement with previous lab and field studies that have suggested that fault slip rate plays a key role in regulating seismic and acoustic properties (Bletery & Nocquet, 2020; Bolton et al., 2020; Dresen et al., 2020; Thomas et al., 2016; 2021; Wech and Bartlow, 2014).

### 4.3 Possible Connections to Tectonic Faulting

The data presented here suggest that slow and fast laboratory earthquakes radiate measurable high‐frequency energy that extends well‐beyond their geodetic corner frequency. These measurements are made possible by the experimental setup and the lack of a strong attenuation barrier from source to receiver. Understanding how these measurements and results connect to tectonic faulting is a challenging problem due to the vast differences in scale and measurements. However, we attempt to connect our data to field observations by considering the relative differences in bandwidth between the geodetic corner frequency and high‐frequency band (100–500 kHz) mentioned above. This comparison is a simplified view and should be taken with caution due to the caveats identified above. In particular, the high‐frequency radiation that we measure is in same bandwidth as the center frequency of our acoustic transducers and spectra have not been corrected for instrument response effects. It is entirely possible that the seismic radiation emanating from slow slip is broadband and we are just detecting it in the 100–500 kHz range because that is where our sensors are most sensitive. Therefore, the “apparent” gap between the geodetic corner frequency and the high‐frequency radiation band could disappear if one were to analyze the complete source displacement spectra in the absence of instrument effects.

At first glance the fact that slow laboratory events radiate high‐frequency energy seems contrary to the long‐held notion that slow tectonic earthquakes are depleted in high‐frequency energy (Ito et al., 2007; Obara, 2002; Shelly et al., 2007). However, we argue that our laboratory observations are consistent with this observation if one accounts for the relative differences between the high‐frequency radiation and corner frequency of the slip events. More specifically, the high‐frequency seismic radiation we measure for the slow slip events in this study is ∼4‐5 orders of magnitude larger than the geodetic corner frequency of the slow events (Figures 2 and 3). The tectonic equivalent of this phenomena is a multi‐day slow slip event and tectonic tremor (Beroza & Ide, 2011; Ide et al., 2007; Thomas et al., 2016). In this case, the slip duration (inverse of corner frequency) of a multi‐day slow slip event is 4‐5 orders of magnitude larger than the slip duration of individual tectonic tremor events (0.1–1s). If this comparison is indeed valid, then our data are consistent with field observations of slow tectonic earthquakes and the high‐frequency signature of our slow slip events is not surprising. In addition, our hypothesis that high‐frequency radiation is regulated by fault slip rate is consistent with observations found in Cascadia showing a positive correlation between slip rate and tremor activity (Bletery & Nocquet, 2020; Wech & Bartlow, 2014). Therefore, our seismic measurements could have important implications for understanding slow slip along crustal fault zones.

The fast events measured in this study have a high‐frequency band that is ∼2‐3 orders of magnitude larger than their geodetic corner frequency (Figures 2 and 3). To our knowledge, there are no field‐based studies that have quantified high‐frequency radiation that extends this far above the measured corner frequency, namely due to the bandlimited nature of the data and/or recording instrument (e.g., Baltay et al., 2010). However, the emerging trend of dense nodal array deployments with excellent station coverage in the near‐source region may permit a more robust and comprehensive analysis of high‐frequency energy characteristics for small and large earthquakes (Catchings et al., 2020; Fan & McGuire, 2018; Kemna et al., 2020; Trugman et al., 2021). Nevertheless, our laboratory data indicate that measurable high‐frequency energy exists well‐beyond the corner frequency and could have important implications for understanding the mode of faulting.

## 5 Conclusion

We analyze AE data associated with a full continuum of slip behaviors, ranging from stable‐sliding to fast‐dynamic stick‐slip. We analyze this full range of lab earthquakes and find systematic differences and similarities in the seismic radiation properties. We demonstrate that a continuum of slip modes, ranging from creep to fast laboratory earthquakes radiate high‐frequency energy between 100 and 500 kHz. We high‐pass filter the AE signals and show that the peak amplitudes of the high‐frequency pulses scale systematically with fault slip velocity. The high‐frequency signals radiated during failure are composed of a myriad of discrete AEs, which most likely represent the failure of tiny fault patches. The high‐frequency characteristics of slow and fast lab stick‐slip events seem to follow different scaling relationships. We augment our stick‐slip experiments with data from stable sliding experiments and demonstrate that high‐frequency energy is modulated by fault slip rate and likely originates from the breaking and sliding of grain contact junctions. Taken together our results suggest that slow and fast laboratory earthquakes radiate detectable high‐frequency energy and variations in attenuation and a lack of high‐resolution measurements near the fault zone could impede these observations in the laboratory and in the field.

## Supporting information

Supporting Information S1

## Data Availability

All data used in this study were collected at The Pennsylvania State University Rock and Sediment Mechanics Laboratory and are publicly available on PSU scholarsphere via the following link: https://scholarsphere.psu.edu/resources/1bdb3598-ba20-44b6-97d4-ea9b77ceb43f.

## References

[jgrb55682-bib-0001] Aben, F. M. , & Brantut, N. (2021). Dilatancy stabilises shear failure in rock. Earth and Planetary Science Letters, 574, 117174. 10.1016/j.epsl.2021.117174

[jgrb55682-bib-0002] Abercrombie, R. , Trugman, D. , Shearer, P. , Chen, X. , Zhang, J. , Pennington, C. , et al. (2021). Does earthquake stress drop increase with Depth in the crust? 10.1002/essoar.10506989.1

[jgrb55682-bib-0003] Aki, K. (1967). Scaling law of seismic spectrum. Journal of geophysical research, 72(4), 1217–1231. 10.1029/jz072i004p01217

[jgrb55682-bib-0004] Baltay, A. , Prieto, G. , & Beroza, G. C. (2010). Radiated seismic energy from coda measurements and no scaling in apparent stress with seismic moment. *Journal of Geophysical Research*, 115(B8). 10.1029/2009jb006736

[jgrb55682-bib-0005] Bedford, J. D. , & Faulkner, D. R. (2021). The role of grain size and effective normal stress on localization and the frictional stability of simulated quartz gouge. Geophysical Research Letters, 48(7), e2020GL092023. 10.1029/2020gl092023

[jgrb55682-bib-0006] Behr, W. M. , & Bürgmann, R. (2020). Whats down there? The structures, materials and environment of deep‐seated tremor and slip. 10.1098/rsta.2020.0218 PMC7898123 33517877

[jgrb55682-bib-0007] Ben‐David, O. , Cohen, G. , & Fineberg, J. (2010). The dynamics of the onset of frictional slip. Science, 330(6001), 211–214. 10.1126/science.1194777 20929771

[jgrb55682-bib-0008] Ben‐Zion, Y. , & Zaliapin, I. (2020). Localization and coalescence of seismicity before large earthquakes. Geophysical Journal International, 223(1), 561–583. 10.1093/gji/ggaa315

[jgrb55682-bib-0009] Beroza, G. C. , & Ide, S. (2011). Slow earthquakes and nonvolcanic tremor. Annual Review of Earth and Planetary Sciences, 39, 271–296. 10.1146/annurev-earth-040809-152531

[jgrb55682-bib-0010] Blanke, A. , Kwiatek, G. , Goebel, T. H. , Bohnhoff, M. , & Dresen, G. (2021). Stress drop–magnitude dependence of acoustic emissions during laboratory stick‐slip. Geophysical Journal International, 224(2), 1371–1380.

[jgrb55682-bib-0011] Bletery, Q. , & Nocquet, J. M. (2020). Slip bursts during coalescence of slow slip events in Cascadia. Nature Communications, 11(1), 1–6. 10.1038/s41467-020-15494-4 PMC7195424 32358488

[jgrb55682-bib-0012] Bolton, D. C. , Shreedharan, S. , Rivière, J. , & Marone, C. (2020). Acoustic energy release during the laboratory seismic cycle: Insights on laboratory earthquake precursors and prediction. Journal of Geophysical Research: Solid Earth, 125, e2019JB018975. 10.1029/2019jb018975 PMC7685124 33282618

[jgrb55682-bib-0013] Bolton, D. C. , Shreedharan, S. , Rivière, J. , & Marone, C. (2021). Frequency‐magnitude statistics of laboratory foreshocks vary with shear velocity, fault slip rate, and shear stress. *Journal of Geophysical Research: Solid Earth*, e2021JB022175. 10.1029/2021jb022175 PMC9286047 35865108

[jgrb55682-bib-0014] Boore, D. M. (1983). Stochastic simulation of high‐frequency ground motions based on seismological models of the radiated spectra. Bulletin of the Seismological Society of America, 73(6A), 1865–1894.

[jgrb55682-bib-0015] Bostock, M. G. , Thomas, A. M. , Rubin, A. M. , & Christensen, N. I. (2017). On corner frequencies, attenuation, and low‐frequency earthquakes. Journal of Geophysical Research: Solid Earth, 122(1), 543–557. 10.1002/2016jb013405

[jgrb55682-bib-0016] Bostock, M. G. , Thomas, A. M. , Savard, G. , Chuang, L. , & Rubin, A. M. (2015). Magnitudes and moment‐duration scaling of low‐frequency earthquakes beneath southern Vancouver Island. Journal of Geophysical Research: Solid Earth, 120(9), 6329–6350. 10.1002/2015jb012195

[jgrb55682-bib-0017] Brune, J. N. (1970). Tectonic stress and the spectra of seismic shear waves from earthquakes. Journal of geophysical research, 75(26), 4997–5009. 10.1029/jb075i026p04997

[jgrb55682-bib-0018] Catchings, R. D. , Goldman, M. R. , Steidl, J. H. , Chan, J. H. , Allam, A. A. , Criley, C. J. , et al. (2020). Nodal seismograph recordings of the 2019 ridgecrest earthquake sequence. Seismological Research Letters, 91(6), 3622–3633. 10.1785/0220200203

[jgrb55682-bib-0019] Dresen, G. , Kwiatek, G. , Goebel, T. , & Ben‐Zion, Y. (2020). Seismic and aseismic preparatory processes before large stick–slip failure. Pure and Applied Geophysics, 177(12), 5741–5760. 10.1007/s00024-020-02605-x

[jgrb55682-bib-0020] Fan, W. , & McGuire, J. J. (2018). Investigating microearthquake finite source attributes with IRIS community wavefield demonstration experiment in Oklahoma. Geophysical Journal International, 214(2), 1072–1087. 10.1093/gji/ggy203

[jgrb55682-bib-0021] Fan, Y. , Gu, F. , & Ball, A. (2010). Modelling acoustic emissions generated by sliding friction. Wear, 268(5–6), 811–815. 10.1016/j.wear.2009.12.010

[jgrb55682-bib-0022] Frank, W. B. , & Brodsky, E. E. (2019). Daily measurement of slow slip from low‐frequency earthquakes is consistent with ordinary earthquake scaling. Science Advances, 5(10), eaaw9386. 10.1126/sciadv.aaw9386 31616786 PMC6774729

[jgrb55682-bib-0023] Goebel, T. H. , Kwiatek, G. , Becker, T. W. , Brodsky, E. E. , & Dresen, G. (2017). What allows seismic events to grow big? Insights from b‐value and fault roughness analysis in laboratory stick‐slip experiments. Geology, 45(9), 815–818. 10.1130/g39147.1

[jgrb55682-bib-0024] Gomberg, J. , Creager, K. , Sweet, J. , Vidale, J. , Ghosh, A. , & Hotovec, A. (2012). Earthquake spectra and near‐source attenuation in the Cascadia subduction zone. *Journal of Geophysical Research*, 117(B5). 10.1029/2011jb009055

[jgrb55682-bib-0025] Goodfellow, S. D. , & Young, R. P. (2014). A laboratory acoustic emission experiment under in situ conditions. Geophysical Research Letters, 41(10), 3422–3430. 10.1002/2014gl059965

[jgrb55682-bib-0026] Hanks, T. C. , & McGuire, R. K. (1981). The character of high‐frequency strong ground motion. Bulletin of the Seismological Society of America, 71(6), 2071–2095. 10.1785/bssa0710062071

[jgrb55682-bib-0027] Hanks, T. C. , & Wyss, M. (1972). The use of body‐wave spectra in the determination of seismic‐source parameters. Bulletin of the Seismological Society of America, 62(2), 561–589. 10.1785/bssa0620020561

[jgrb55682-bib-0028] Ide, S. , & Beroza, G. C. (2001). Does apparent stress vary with earthquake size? Geophysical Research Letters, 28(17), 3349–3352. 10.1029/2001gl013106

[jgrb55682-bib-0029] Ide, S. , Beroza, G. C. , Shelly, D. R. , & Uchide, T. (2007). A scaling law for slow earthquakes. Nature, 447(7140), 76–79. 10.1038/nature05780 17476265

[jgrb55682-bib-0030] Ito, Y. , Obara, K. , Shiomi, K. , Sekine, S. , & Hirose, H. (2007). Slow earthquakes coincident with episodic tremors and slow slip events. Science, 315(5811), 503–506. 10.1126/science.1134454 17138867

[jgrb55682-bib-0031] Kao, H. , Shan, S. J. , Dragert, H. , Rogers, G. , Cassidy, J. F. , & Ramachandran, K. (2005). A wide depth distribution of seismic tremors along the northern Cascadia margin. Nature, 436(7052), 841–844. 10.1038/nature03903 16094366

[jgrb55682-bib-0032] Kaproth, B. M. , & Marone, C. (2013). Slow earthquakes, preseismic velocity changes, and the origin of slow frictional stick‐slip. Science, 341(6151), 1229–1232. 10.1126/science.1239577 23950495

[jgrb55682-bib-0033] Karner, S. L. , & Marone, C. (2000). Effects of loading rate and normal stress on stress drop and stick‐slip recurrence interval (Vol. 120, pp. 187–198). Geophysical Monograph‐American Geophysical Union. 10.1029/gm120p0187

[jgrb55682-bib-0034] Kato, A. , Obara, K. , Igarashi, T. , Tsuruoka, H. , Nakagawa, S. , & Hirata, N. (2012). Propagation of slow slip leading up to the 2011 Mw 9.0 Tohoku‐Oki earthquake. Science, 335(6069), 705–708. 10.1126/science.1215141 22267578

[jgrb55682-bib-0035] Kemna, K. B. , Peña Castro, A. F. , Harrington, R. M. , & Cochran, E. S. (2020). Using a large‐n seismic array to explore the robustness of spectral estimations. Geophysical Research Letters, 47(21), e2020GL089342. 10.1029/2020gl089342

[jgrb55682-bib-0036] Kenigsberg, A. R. , Rivière, J. , Marone, C. , & Saffer, D. M. (2019). The effects of shear strain, fabric, and porosity evolution on elastic and mechanical properties of clay‐rich fault gouge. Journal of Geophysical Research: Solid Earth, 124(11), 10968–10982. 10.1029/2019jb017944

[jgrb55682-bib-0037] Kenigsberg, A. R. , Rivière, J. , Marone, C. , & Saffer, D. M. (2020). Evolution of elastic and mechanical properties during fault shear: The roles of clay content, fabric development, and porosity. Journal of Geophysical Research: Solid Earth, 125(3), e2019JB018612. 10.1029/2019jb018612

[jgrb55682-bib-0038] Latour, S. , Schubnel, A. , Nielsen, S. , Madariaga, R. , & Vinciguerra, S. (2013). Characterization of nucleation during laboratory earthquakes. Geophysical Research Letters, 40(19), 5064–5069. 10.1002/grl.50974

[jgrb55682-bib-0039] Leeman, J. R. , Marone, C. , & Saffer, D. M. (2018). Frictional mechanics of slow earthquakes. Journal of Geophysical Research: Solid Earth, 123(9), 7931–7949. 10.1029/2018jb015768

[jgrb55682-bib-0040] Leeman, J. R. , Saffer, D. M. , Scuderi, M. M. , & Marone, C. (2016). Laboratory observations of slow earthquakes and the spectrum of tectonic fault slip modes. Nature Communications, 7(1), 1–6. 10.1038/ncomms11104 PMC4821871 27029996

[jgrb55682-bib-0041] Lockner, D. , Byerlee, J. D. , Kuksenko, V. , Ponomarev, A. , & Sidorin, A. (1991). Quasi‐static fault growth and shear fracture energy in granite. Nature, 350(6313), 39–42. 10.1038/350039a0

[jgrb55682-bib-0042] Madariaga, R. (1977). High‐frequency radiation from crack (stress drop) models of earthquake faulting. Geophysical Journal International, 51(3), 625–651. 10.1111/j.1365-246x.1977.tb04211.x

[jgrb55682-bib-0043] Madariaga, R. (1983). High frequency radiation from dynamic earthquake. Annals of Geophysics, 1, 17.

[jgrb55682-bib-0044] Mair, K. , Frye, K. M. , & Marone, C. (2002). Influence of grain characteristics on the friction of granular shear zones. Journal of Geophysical Research, 107(B10), ECV–4. 10.1029/2001jb000516

[jgrb55682-bib-0045] Mair, K. , & Marone, C. (1999). Friction of simulated fault gouge for a wide range of velocities and normal stresses. Journal of Geophysical Research, 104(B12), 28899–28914. 10.1029/1999jb900279

[jgrb55682-bib-0046] Marone, C. (1998). Laboratory‐derived friction laws and their application to seismic faulting. Annual Review of Earth and Planetary Sciences, 26(1), 643–696. 10.1146/annurev.earth.26.1.643

[jgrb55682-bib-0047] Marone, C. , & Kilgore, B. (1993). Scaling of the critical slip distance for seismic faulting with shear strain in fault zones. Nature, 362(6421), 618–621. 10.1038/362618a0

[jgrb55682-bib-0048] Marone, C. , Raleigh, C. B. , & Scholz, C. H. (1990). Frictional behavior and constitutive modeling of simulated fault gouge. Journal of Geophysical Research, 95(B5), 7007–7025. 10.1029/jb095ib05p07007

[jgrb55682-bib-0049] Marty, S. , Passelègue, F. X. , Aubry, J. , Bhat, H. S. , Schubnel, A. , & Madariaga, R. (2019). Origin of high‐frequency radiation during laboratory earthquakes. Geophysical Research Letters, 46(7), 3755–3763. 10.1029/2018gl080519

[jgrb55682-bib-0050] McBeck, J. , Ben‐Zion, Y. , & Renard, F. (2021). Volumetric and shear strain localization throughout triaxial compression experiments on rocks (p. 229181). Tectonophysics.

[jgrb55682-bib-0051] McBeck, J. , Kobchenko, M. , Hall, S. A. , Tudisco, E. , Cordonnier, B. , Meakin, P. , & Renard, F. (2018). Investigating the onset of strain localization within anisotropic shale using digital volume correlation of time‐resolved X‐ray microtomography images. Journal of Geophysical Research: Solid Earth, 123(9), 7509–7528. 10.1029/2018jb015676

[jgrb55682-bib-0052] McLaskey, G. C. , & Glaser, S. D. (2011). Micromechanics of asperity rupture during laboratory stick slip experiments. *Geophysical Research Letters*, 38(12). 10.1029/2011gl047507

[jgrb55682-bib-0053] McLaskey, G. C. , & Lockner, D. A. (2014). Preslip and cascade processes initiating laboratory stick slip. Journal of Geophysical Research: Solid Earth, 119(8), 6323–6336. 10.1002/2014jb011220

[jgrb55682-bib-0054] McLaskey, G. C. , Lockner, D. A. , Kilgore, B. D. , & Beeler, N. M. (2015). A robust calibration technique for acoustic emission systems based on momentum transfer from a ball drop. Bulletin of the Seismological Society of America, 105(1), 257–271. 10.1785/0120140170

[jgrb55682-bib-0055] McLaskey, G. C. , Thomas, A. M. , Glaser, S. D. , & Nadeau, R. M. (2012). Fault healing promotes high‐frequency earthquakes in laboratory experiments and on natural faults. Nature, 491(7422), 101–104. 10.1038/nature11512 23128232

[jgrb55682-bib-0056] Mclaskey, G. C. , & Yamashita, F. (2017). Slow and fast ruptures on a laboratory fault controlled by loading characteristics. Journal of Geophysical Research: Solid Earth, 122(5), 3719–3738. 10.1002/2016jb013681

[jgrb55682-bib-0057] Michel, S. , Gualandi, A. , & Avouac, J. P. (2019). Similar scaling laws for earthquakes and Cascadia slow‐slip events. Nature, 574(7779), 522–526. 10.1038/s41586-019-1673-6 31645722

[jgrb55682-bib-0058] Miller, P. K. , Saffer, D. M. , Abers, G. A. , Shillington, D. J. , Bécel, A. , Li, J. , & Bate, C. (2021). P‐and S‐wave velocities of exhumed metasediments from the Alaskan subduction zone: Implications for the in situ conditions along the megathrust. Geophysical Research Letters, 48(20), e2021GL094511. 10.1029/2021gl094511

[jgrb55682-bib-0059] Obara, K. (2002). Nonvolcanic deep tremor associated with subduction in southwest Japan. Science, 296, 1679–1681. 10.1126/science.1070378 12040191

[jgrb55682-bib-0060] Obara, K. , & Kato, A. (2016). Connecting slow earthquakes to huge earthquakes. Science, 353(6296), 253–257. 10.1126/science.aaf1512 27418504

[jgrb55682-bib-0061] Oth, A. , Miyake, H. , & Bindi, D. (2017). On the relation of earthquake stress drop and ground motion variability. Journal of Geophysical Research: Solid Earth, 122(7), JB014026. 10.1002/2017JB014026

[jgrb55682-bib-0062] Passelègue, F. X. , Almakari, M. , Dublanchet, P. , Barras, F. , Fortin, J. , & Violay, M. (2020). Initial effective stress controls the nature of earthquakes. Nature Communications, 11(1), 1–8. 10.1038/s41467-020-18937-0 PMC7552404 33046700

[jgrb55682-bib-0063] Passelègue, F. X. , Schubnel, A. , Nielsen, S. , Bhat, H. S. , Deldicque, D. , & Madariaga, R. (2016). Dynamic rupture processes inferred from laboratory microearthquakes. Journal of Geophysical Research: Solid Earth, 121(6), 4343–4365. 10.1002/2015jb012694

[jgrb55682-bib-0064] Peng, Z. , & Gomberg, J. (2010). An integrated perspective of the continuum between earthquakes and slow‐slip phenomena. Nature Geoscience, 3(9), 599–607. 10.1038/ngeo940

[jgrb55682-bib-0065] Prieto, G. A. , Shearer, P. M. , Vernon, F. L. , & Kilb, D. (2004). Earthquake source scaling and self‐similarity estimation from stacking P and S spectra. *Journal of Geophysical Research*, 109(B8). 10.1029/2004jb003084

[jgrb55682-bib-0066] Renard, F. , McBeck, J. , Cordonnier, B. , Zheng, X. , Kandula, N. , Sanchez, J. R. , et al. (2019). Dynamic in situ three‐dimensional imaging and digital volume correlation analysis to quantify strain localization and fracture coalescence in sandstone. Pure and Applied Geophysics, 176(3), 1083–1115. 10.1007/s00024-018-2003-x

[jgrb55682-bib-0067] Rivière, J. , Lv, Z. , Johnson, P. A. , & Marone, C. (2018). Evolution of b‐value during the seismic cycle: Insights from laboratory experiments on simulated faults. Earth and Planetary Science Letters, 482, 407–413. 10.1016/j.epsl.2017.11.036

[jgrb55682-bib-0068] Rogers, G. , & Dragert, H. (2003). Episodic tremor and slip on the Cascadia subduction zone: The chatter of silent slip. Science, 300(5627), 1942–1943. 10.1126/science.1084783 12738870

[jgrb55682-bib-0069] Rubinstein, J. L. , Vidale, J. E. , Gomberg, J. , Bodin, P. , Creager, K. C. , & Malone, S. D. (2007). Non‐volcanic tremor driven by large transient shear stresses. Nature, 448(7153), 579–582. 10.1038/nature06017 17671500

[jgrb55682-bib-0070] Savage, J. C. (1972). Relation of corner frequency to fault dimensions. Journal of geophysical research, 77(20), 3788–3795. 10.1029/jb077i020p03788

[jgrb55682-bib-0071] Scholz, C. H. (1968). The frequency‐magnitude relation of microfracturing in rock and its relation to earthquakes. Bulletin of the Seismological Society of America, 58(1), 399–415. 10.1785/bssa0580010399

[jgrb55682-bib-0072] Scholz, C. H. (2019). The mechanics of earthquakes and faulting. Cambridge University Press. 10.1126/science.250.4988.1758-a 17734719

[jgrb55682-bib-0073] Scuderi, M. M. , Collettini, C. , Viti, C. , Tinti, E. , & Marone, C. (2017). Evolution of shear fabric in granular fault gouge from stable sliding to stick slip and implications for fault slip mode. Geology, 45(8), 731–734. 10.1130/g39033.1

[jgrb55682-bib-0074] Scuderi, M. M. , Marone, C. , Tinti, E. , Di Stefano, G. , & Collettini, C. (2016). Precursory changes in seismic velocity for the spectrum of earthquake failure modes. Nature Geoscience, 9(9), 695–700. 10.1038/ngeo2775 PMC5010128 27597879

[jgrb55682-bib-0075] Scuderi, M. M. , Tinti, E. , Cocco, M. , & Collettini, C. (2020). The role of shear fabric in controlling breakdown processes during laboratory slow‐slip events. Journal of Geophysical Research: Solid Earth, 125(11), e2020JB020405. 10.1029/2020jb020405

[jgrb55682-bib-0076] Segall, P. , & Bradley, A. M. (2012). Slow‐slip evolves into megathrust earthquakes in 2D numerical simulations. *Geophysical Research Letters*, 39(18). 10.1029/2012gl052811

[jgrb55682-bib-0077] Shelly, D. R. , Beroza, G. C. , & Ide, S. (2007). Non‐volcanic tremor and low‐frequency earthquake swarms. Nature, 446(7133), 305–307. 10.1038/nature05666 17361180

[jgrb55682-bib-0078] Shreedharan, S. , Bolton, D. C. , Rivière, J. , & Marone, C. (2020). Preseismic fault creep and elastic wave amplitude precursors scale with lab earthquake magnitude for the continuum of tectonic failure modes. *Geophysical Research Letters*, 46. 10.1029/2020gl086986

[jgrb55682-bib-0079] Socquet, A. , Valdes, J. P. , Jara, J. , Cotton, F. , Walpersdorf, A. , Cotte, N. , et al. (2017). An 8 month slow slip event triggers progressive nucleation of the 2014 Chile megathrust. Geophysical Research Letters, 44(9), 4046–4053. 10.1002/2017gl073023

[jgrb55682-bib-0080] Supino, M. , Poiata, N. , Festa, G. , Vilotte, J. P. , Satriano, C. , & Obara, K. (2020). Self‐similarity of low‐frequency earthquakes. Scientific Reports, 10(1), 1–9. 10.1038/s41598-020-63584-6 32300164 PMC7162910

[jgrb55682-bib-0081] Thomas, A. M. , Beroza, G. C. , & Shelly, D. R. (2016). Constraints on the source parameters of low‐frequency earthquakes on the San Andreas Fault. Geophysical Research Letters, 43(4), 1464–1471. 10.1002/2015gl067173

[jgrb55682-bib-0082] Tinti, E. , Scuderi, M. , Scognamiglio, L. , Di Stefano, G. , Marone, C. , & Collettini, C. (2016). On the evolution of elastic properties during laboratory stick‐slip experiments spanning the transition from slow slip to dynamic rupture. Journal of Geophysical Research: Solid Earth, 121(12), 8569–8594. 10.1002/2016jb013545

[jgrb55682-bib-0083] Trugman, D. T. , Chu, S. X. , & Tsai, V. C. (2021). Earthquake source complexity controls the frequency dependence of near‐source radiation patterns. Geophysical Research Letters, 48(17), e2021GL095022. 10.1029/2021gl095022

[jgrb55682-bib-0084] Trugman, D. T. , & Shearer, P. M. (2018). Strong correlation between stress drop and peak ground acceleration for recent M 1–4 earthquakes in the San Francisco Bay area. Bulletin of the Seismological Society of America, 108(2), 929–945. 10.1785/0120170245

[jgrb55682-bib-0085] Tsai, V. C. , & Hirth, G. (2020). Elastic impact consequences for high‐frequency earthquake ground motion. Geophysical Research Letters, 47(5), e2019GL086302. 10.1029/2019gl086302

[jgrb55682-bib-0086] Tsai, V. C. , Hirth, G. , Trugman, D. T. , & Chu, S. X. (2021). Impact versus frictional earthquake models for high‐frequency radiation in Complex fault zones. *Journal of Geophysical Research: Solid Earth*, e2021JB022313. 10.1029/2021jb022313

[jgrb55682-bib-0087] Veedu, D. M. , & Barbot, S. (2016). The Parkfield tremors reveal slow and fast ruptures on the same asperity. Nature, 532(7599), 361–365. 10.1038/nature17190 27042936

[jgrb55682-bib-0088] Wech, A. G. , & Bartlow, N. M. (2014). Slip rate and tremor Genesis in Cascadia. Geophysical Research Letters, 41(2), 392–398. 10.1002/2013gl058607

[jgrb55682-bib-0089] Wu, B. S. , & McLaskey, G. C. (2018). Broadband calibration of acoustic emission and ultrasonic sensors from generalized ray theory and finite element models. Journal of Nondestructive Evaluation, 37(1), 1–16. 10.1007/s10921-018-0462-8

[jgrb55682-bib-0090] Wu, B. S. , & McLaskey, G. C. (2019). Contained laboratory earthquakes ranging from slow to fast. Journal of Geophysical Research: Solid Earth, 124(10), 10270–10291. 10.1029/2019jb017865

[jgrb55682-bib-0091] Zigone, D. , Voisin, C. , Larose, E. , Renard, F. , & Campillo, M. (2011). Slip acceleration generates seismic tremor like signals in friction experiments. *Geophysical Research Letters*, 38(1). 10.1029/2010gl045603

