# Supplementary material for: The High‐Frequency Signature of Slow and Fast Laboratory Earthquakes
Source: J Geophys Res Solid Earth. 2022 Jun 7;127(6):e2022JB024170. doi: 10.1029/2022JB024170 (PMC9287021; doi:10.1029/2022JB024170)
Supplement: Supplementary file 1 — Supporting Information S1 [file JGRB-127-0-s001.docx]

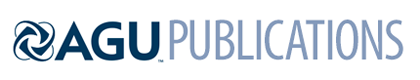


**Journal of Geophysical Research: Solid Earth**

**Supporting Information for:**

**The high-frequency signature of slow and fast laboratory earthquakes**

David C. Bolton^1*^, Srisharan Shreedharan^1^, Greg McLaskey^2^, Jacques Riviere^3^, Parisa Shokouhi^3^, Daniel T. Trugman^4^, and Chris Marone^5,6^

^1^ University of Texas Institute for Geophysics, Austin, TX

^2^Department of Civil and Environmental Engineering, Cornell University, Ithaca, NY

^3^Department of Engineering Science and Mechanics Pennsylvania State University, University Park, Pennsylvania

^4^Nevada Seismological Laboratory, University of Nevada, Reno, Nevada

^5^Department of Geosciences, Pennsylvania State University, University Park, Pennsylvania

^6^Dipartimento di Scienze della Terra, La Sapienza Universita di Roma, Italy

*Corresponding author: David C. Bolton ([chasbolton19@gmail.com)](mailto:chasbolton19@gmail.com))

**Contents of this File:**

Figures S1-S4

**Introduction:**

This supporting information contains figures of amplitude spectra of acoustic traces derived from the co-seismic slip phase. We show how the hydraulic power-supply affects data in the time-domain and frequency-domain. We also quantify the seismic moment, peak friction, and recurrence interval of seismic cycles and document how these properties are modulated by normal stress.


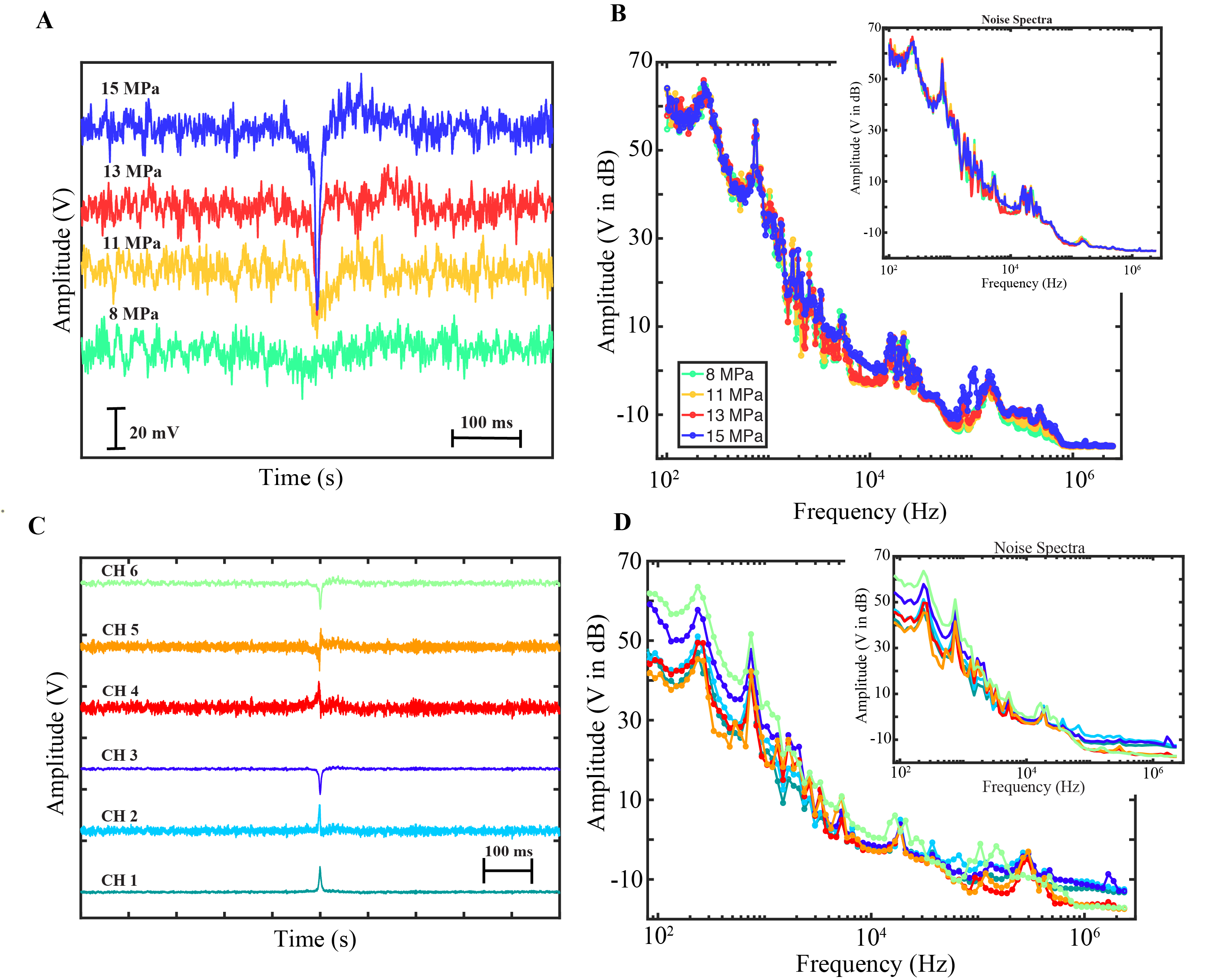


**Figure S1**. **A** Time-domain signals from channel 6 during the co-seismic slip phase. AE signals are from Experiment p5435 (see Figure 3 in main text) and represent different slip events compared to those plotted in Figure 3 of the main text. In general, the time-domain signals look similar to those plotted in Figure 3 of the main-text. **B** Spectra of events shown in A. The spectra have identical shapes to those in Figure 3. However, the fast events (13 and 15 MPa) have identical amplitudes at low frequencies compared to the slow events. In contrast, the fast events in Figure 3 have slightly higher amplitudes at lower frequencies, relative to the slow events. These differences arise from the nature of the time-domain signals and the poor SNR (see Figure 3 in the main text). Inset shows noise traces for data at each normal stress. **C** Time-domain signals for the 15 MPa event shown in A. **D** Amplitude spectra for the traces shown in C. The spectral characteristics are approximately the same across the network. Inset shows noise spectra.

**Figure S2. A-B.** Raw-acoustic traces (2s long) without (A) and with (B) the hydraulic power supply turned on. Note the significant increase in noise due to the hydraulic power supply. **C.** Average spectra of the traces shown in A and B. Here, we average the spectra for all channels in A and B, respectively. The hydraulic power supply contaminates the acoustic signals with noise for frequencies < 10 kHz.


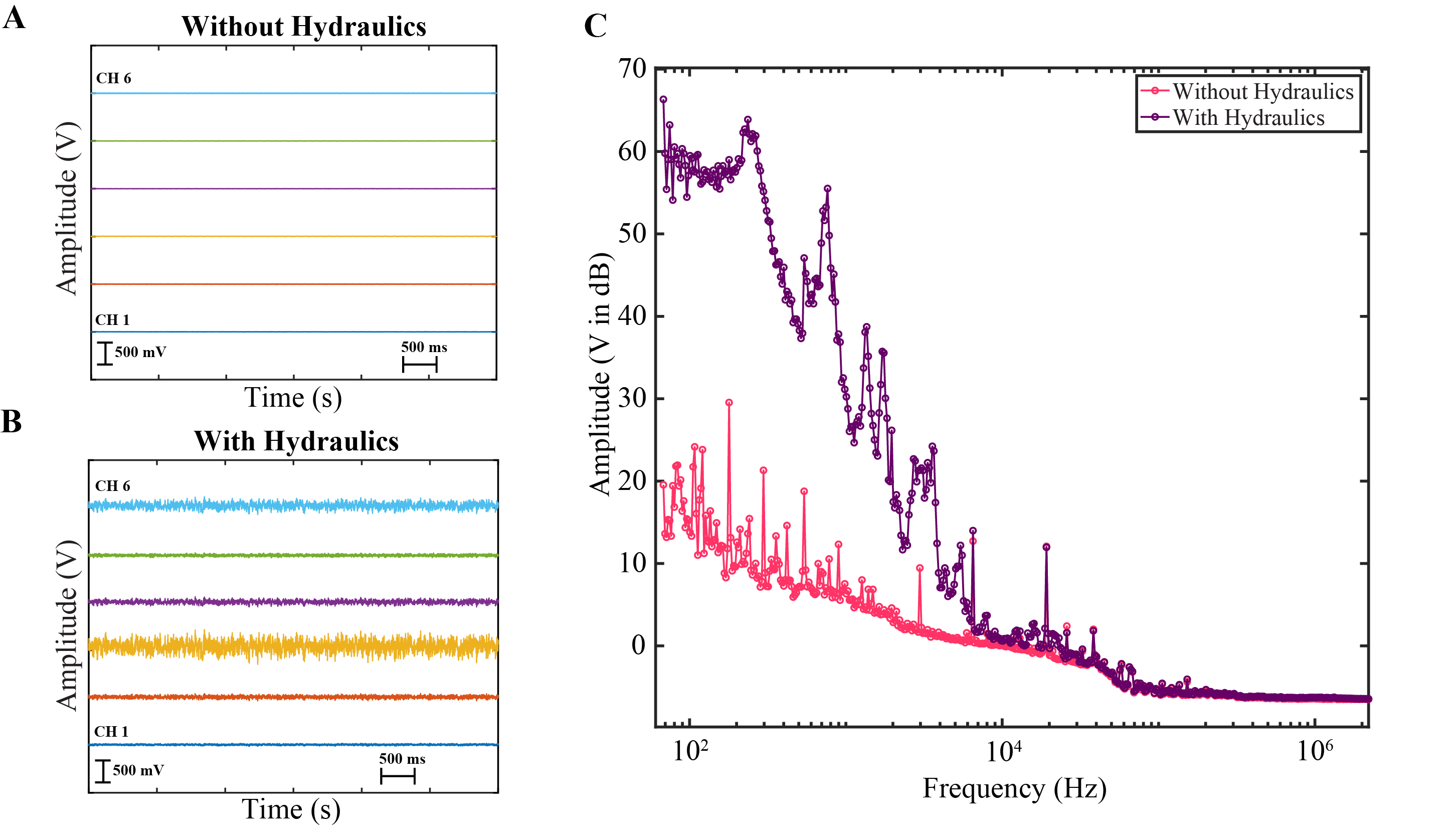


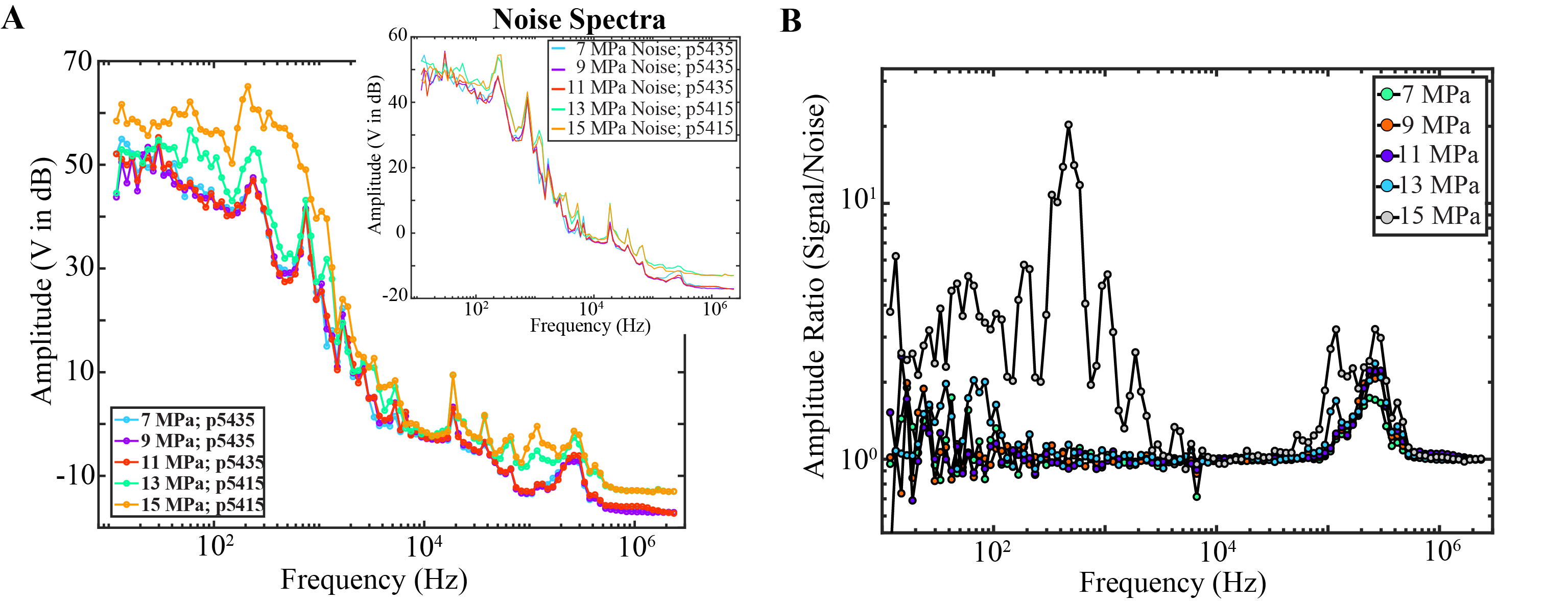


**Figure S3**. **A.** Amplitude spectra from acoustic traces in Figure 3 of the main text. Curves are color coded according to their respective trace. The spectra are essentially the same for the slow events between 7-11 MPa; fast events at 13 and 15 MPa show a modest increase in amplitude at low frequencies (<1000 Hz) and at high-frequencies (>= 10 kHz). Inset shows noise spectra from 2s long traces derived from the initial stages of the seismic cycle. **B.** Signal to noise ratios derived from panel A. Slow events (7-11 MPa) have poor SNR across most of their bandwidth, with values slightly higher than 1 within the 100-500 kHz bandwidth. Fast events (13-15 MPa) have higher SNR for frequencies <10 kHz and between 80-500 kHz.


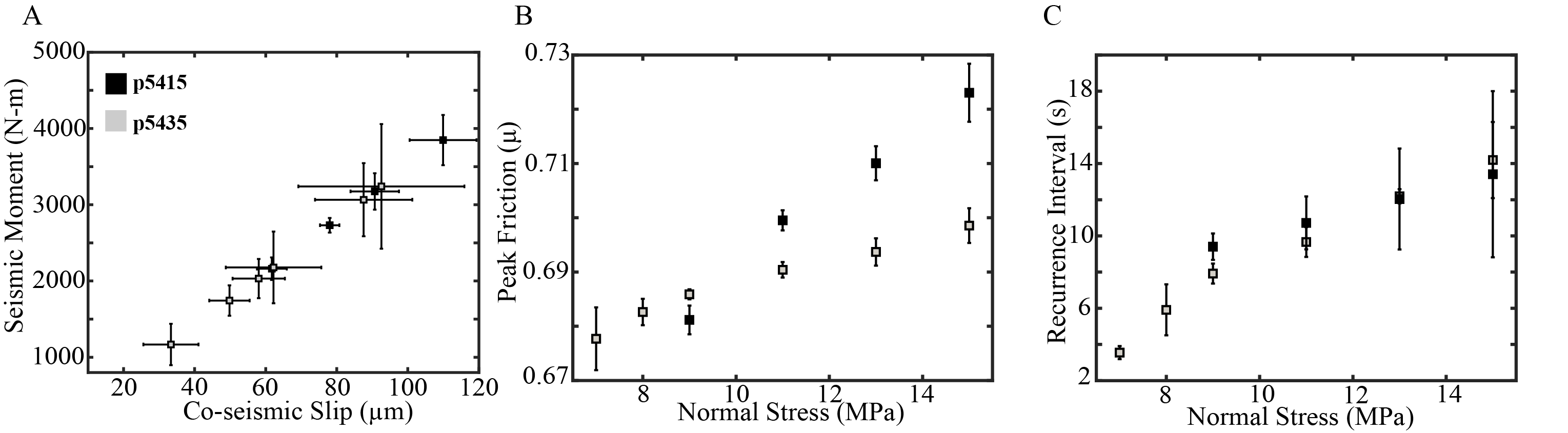


**Figure S4**. **A** Seismic moment as a function of co-seismic slip. Seismic moment is estimated geodetically for one fault and assuming a constant shear modulus of 3.5 GPa. The entire fault slips during the co-seismic slip phase, and thus, the seismic moment is only modulated by variations in co-seismic slip and not area**. B.** Peak friction reached prior to co-seismic slip as a function of normal stress. Peak friction scales systematically with normal stress. **C.** Recurrence interval versus normal stress. Recurrence interval is estimated as the time difference between the peak and minimum shear stress for a given stick-slip cycle. In all plots, symbols represent mean values and error bars represent one standard deviation.
